# Knockdown of *wfs1*, a fly homolog of Wolfram syndrome 1, in the nervous system increases susceptibility to age- and stress-induced neuronal dysfunction and degeneration in *Drosophila*

**DOI:** 10.1371/journal.pgen.1007196

**Published:** 2018-01-22

**Authors:** Yasufumi Sakakibara, Michiko Sekiya, Naoki Fujisaki, Xiuming Quan, Koichi M. Iijima

**Affiliations:** 1 Department of Alzheimer’s Disease Research, Center for Development of Advanced Medicine for Dementia, National Center for Geriatrics and Gerontology, Obu, Aichi, Japan; 2 Department of Experimental Gerontology, Graduate School of Pharmaceutical Sciences, Nagoya City University, 3–1 Tanabe-dori, Mizuho-ku, Nagoya, Japan; Stanford University School of Medicine, UNITED STATES

## Abstract

Wolfram syndrome (WS), caused by loss-of-function mutations in the Wolfram syndrome 1 gene (*WFS1*), is characterized by juvenile-onset diabetes mellitus, bilateral optic atrophy, and a wide spectrum of neurological and psychiatric manifestations. *WFS1* encodes an endoplasmic reticulum (ER)-resident transmembrane protein, and mutations in this gene lead to pancreatic β-cell death induced by high levels of ER stress. However, the mechanisms underlying neurodegeneration caused by *WFS1* deficiency remain elusive. Here, we investigated the role of *WFS1* in the maintenance of neuronal integrity *in vivo* by knocking down the expression of *wfs1*, the *Drosophila* homolog of *WFS1*, in the central nervous system. Neuronal knockdown of *wfs1* caused age-dependent behavioral deficits and neurodegeneration in the fly brain. Knockdown of *wfs1* in neurons and glial cells resulted in premature death and significantly exacerbated behavioral deficits in flies, suggesting that *wfs1* has important functions in both cell types. Although *wfs1* knockdown alone did not promote ER stress, it increased the susceptibility to oxidative stress-, excitotoxicity- or tauopathy-induced behavioral deficits, and neurodegeneration. The glutamate release inhibitor riluzole significantly suppressed premature death phenotypes induced by neuronal and glial knockdown of *wfs1*. This study highlights the protective role of *wfs1* against age-associated neurodegeneration and furthers our understanding of potential disease-modifying factors that determine susceptibility and resilience to age-associated neurodegenerative diseases.

## Author summary

Wolfram syndrome (WS), a neurodegenerative disorder with an autosomal recessive inheritance pattern, has a variable clinical presentation that includes diabetes mellitus, optic atrophy, and a wide spectrum of neurological and psychiatric manifestations. Homozygous mutations in *WFS1* are causative for WS. The prognosis of WS is poor, and most patients die prematurely with respiratory failure due to brain stem atrophy. However, the mechanisms underlying the neurological manifestations of WS remain elusive. In this study, we used the fruit fly *Drosophila* to examine the neurological features of WS by generating genetically modified flies harboring knockdown of *wfs1*, the fly homolog of *WFS1*, in the central nervous system. These flies developed age-dependent behavioral deficits, neurodegeneration and premature death. *wfs1*-deficient flies were vulnerable to various age-related stressors such as oxidative stress and excitotoxicity, and to neurodegeneration caused by Alzheimer’s disease-related toxic proteins. The premature death phenotype in *wfs1*-deficient flies was ameliorated by administration of riluzole, which inhibits glutamate-induced excitotoxicity. This study provides insight into the mechanisms underlying neurodegeneration not only in WS, but also in age-associated neurodegenerative diseases such as Alzheimer’s disease.

## Introduction

Wolfram syndrome (WS; OMIM 222300) is a progressive neurodegenerative disorder characterized by an autosomal recessive inheritance pattern [1]. The clinical manifestations of WS are highly variable and include diabetes insipidus, diabetes mellitus, optic atrophy, and deafness, known as the DIDMOAD syndrome [1]. Juvenile-onset diabetes mellitus and bilateral optic atrophy, which usually emerge by the second decade of life, are the minimal criteria for diagnosis [2,3].

Molecular genetic studies show that 90% of WS patients carry a loss-of-function mutation in the *WFS1* gene [4,5], which encodes an endoplasmic reticulum (ER)-resident transmembrane protein [6,7]. Although the WFS1 protein lacks a distinct functional domain structure, a growing body of evidence indicates that WFS1 regulates the cellular response to ER stress [8–12] as well as intracellular Ca^2+^ homeostasis [13–15]. *WFS1* deletion in mice leads to progressive loss of pancreatic β cells and impaired glucose tolerance induced by high levels of ER stress and apoptosis in β cells [11,16,17]. Thus, the pathogenesis of WS involves chronic ER stress in pancreatic β cells leading to diabetes mellitus [8–11], and WS is considered as a prototype of ER disease [8,13,16]. Common variants in *WFS1* are also associated with the risk of type 2 diabetes [18].

In addition to these cardinal features of the disorder, WS patients exhibit a wide spectrum of neurological and psychiatric manifestations including peripheral neuropathy, epilepsy, cognitive decline, and anxiety [19,20]. Magnetic resonance imaging studies show diffuse and widespread atrophy throughout the brain [21–23], which partly correlates with the major neurological features of the disorder. The prognosis of WS is poor, and most patients die prematurely with severe neurological disabilities at a median age of 30 years, usually from respiratory failure resulting from brain stem atrophy [1,24,25]. Carriers of *WFS1* mutations who are not affected by WS have a 26-fold higher likelihood of psychiatric hospitalization, primarily for depression [26]. Consistent with this, heterozygosity for *WFS1* gene mutations, which is present in up to 1% of the general population, is a significant cause of psychiatric illness in the general population [26–28]. Therefore, elucidating the mechanisms underlying the effects of *WFS1* mutations will advance our understanding of neurological and neurodegenerative diseases and lead to the development of therapeutic strategies to halt the progression of *WFS1*-related disorders.

*Wfs1* knockout mice recapitulate several aspects of the neurological manifestations of WS, including impaired behavioral adaptation to stress [29,30], stress-induced depressive behavior [31], and alterations in visual function [32]. A recent study used a rat model to demonstrate that deletion of exon 5 of the *Wfs1* gene, which results in the loss of 27 amino acids from the Wfs1 protein, caused insulin-dependent diabetes, optic nerve atrophy, and medullary degeneration [33]. However, the mechanisms underlying the neurological manifestations of *WFS1* deficiency remain elusive.

In this study, we used *Drosophila* as a genetic model to analyze the effects of *WFS1* deficiency on neuronal integrity during aging. According to the DIOPT (DRSC Integrative Ortholog Prediction Tool), *Drosophila wfs1* (*CG4917*) is the best homolog of human *WFS1* proteins (DIOPT score 10); wfs1 and WFS1 exhibit 25% identity and 42% similarity in primary amino acid sequence, are similar in size (853 and 890 amino acids, respectively), and have the same nine predicted transmembrane helices (TMHMM Server v. 2.0). Analysis of *wfs1* mutant flies and flies with RNAi-mediated knockdown of *wfs1* in the central nervous system showed that *wfs1* deficiency increased susceptibility to age- and stress-induced neuronal dysfunction and degeneration. Our results also suggest that imbalance in neural activity and vulnerability to oxidative stress are involved in this process.

## Results

### Neuronal knockdown of *wfs1*, a fly homolog of *WFS1*, causes behavioral deficits and neurodegeneration

**Fig 1:**
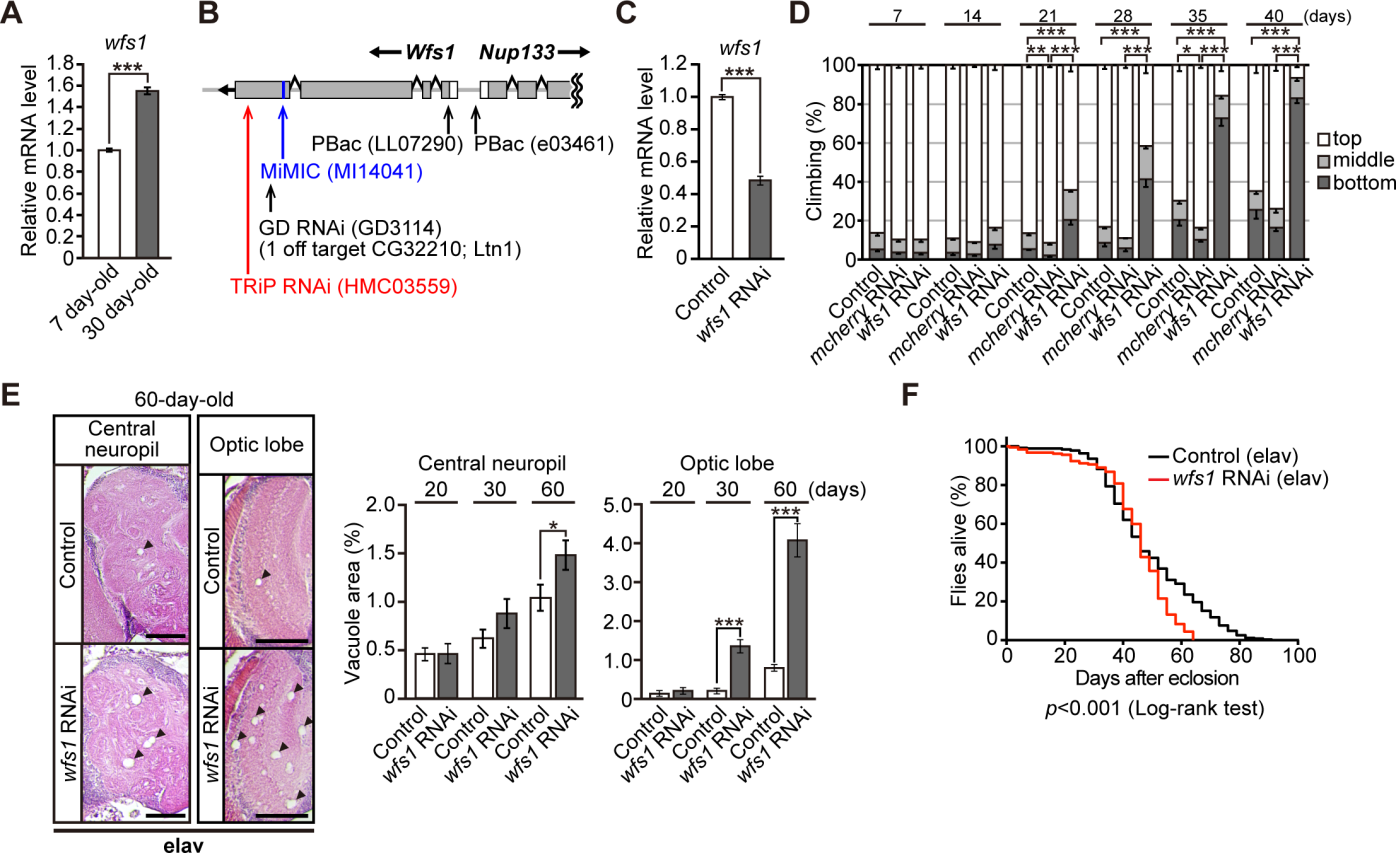
Neuronal knockdown of *wfs1*, a fly homolog of *WFS1*, causes behavioral deficits and neurodegeneration. (**A**) mRNA expression levels of *wfs1* were increased upon aging in the fly brains, as determined by qRT-PCR. n = 4, ***p < 0.001 by Student’s *t*-test. (**B**) A schematic diagram of the *wfs1* RNAi target sites and insertion sites of MiMIC/PiggyBac for *wfs1* mutant lines. (**C**) mRNA levels of *wfs1* in heads of flies carrying the RNAi transgene targeting *wfs1* were analyzed by qRT-PCR. n = 4, ***p < 0.001 by Student’s *t*-test. (**D**) Knockdown of *wfs1* in neurons induced age-dependent locomotor deficits as revealed by climbing assay. Flies carrying the *mcherry* RNAi transgene did not induce the age-dependent locomotor deficits. Average percentages of flies that climbed to the top (white), climbed to the middle (light gray), or stayed at the bottom (dark gray) of the vials. Percentages of flies that stayed at the bottom were subjected to statistical analyses. n = 7 independent experiments, *p < 0.05, **p < 0.01 and ***p < 0.001 by Student’s *t*-test. (**E**) Neuronal knockdown of *wfs1* caused age-dependent neurodegeneration in the central neuropil or optic lobes of fly brain. Representative images show the central neuropil and optic lobe in paraffin-embedded brain section with hematoxylin and eosin (HE) staining from 60-day-old flies. Scale bars: 200 μm. Percentages of vacuole areas (indicated by arrowheads in the images) in central neuropil or optic lobes from 20-, 30-, and 60-day-old flies were analyzed. n = 8–12 hemispheres, *p < 0.05 and ***p < 0.001 by Student’s *t*-test. (**F**) Neuronal knockdown of *wfs1* shortened the lifespan (n = 182, *wfs1* RNAi group or 358, control group). The lifespans of flies were determined by Kaplan-Meier survival analysis with log-rank test and statistical significance was indicated in the figure. Genotypes and ages of flies are described in S1 Table.

In rodent brains, the mRNA levels of *Wfs1* increase during aging [34]. Quantitative PCR (qPCR) analysis showed that the mRNA levels of *Drosophila wfs1*, a fly homolog of human *WFS1*, increased with aging in fly brains (Fig 1A). These results indicated that *WFS1* may play an evolutionarily conserved role in maintaining neuronal integrity during aging.

To elucidate the roles of *WFS1* in neuronal integrity during aging in *Drosophila*, we knocked down *wfs1* in the central nervous system. The GAL4/UAS system was used to induce neuron-specific knockdown of *wfs1* by crossing transgenic flies carrying RNAi targeting *wfs1* (TRiP RNAi, Fig 1B) with flies carrying the pan-neuronal *elav*-GAL4 driver. qPCR confirmed that neuron-specific expression of *wfs1* RNAi significantly reduced *wfs1* mRNA levels in fly brains (Fig 1C).

To determine whether neuron-specific *wfs1* knockdown caused age-dependent neuronal dysfunction, locomotor ability was assessed using the forced climbing assay. Neuronal knockdown of *wfs1* induced age-dependent locomotor deficits starting at 21 days of age (Fig 1D), suggesting that *wfs1* deficiency in neurons is sufficient to induce age-dependent behavioral deficits. In *Drosophila*, brain vacuolation is a morphological hallmark of neurodegeneration [35]. Neuronal knockdown of *wfs1* significantly increased the age-dependent appearance of vacuoles in the central neuropil and optic lobes of the fly brain (Fig 1E) and slightly, but significantly, shortened the lifespan of the flies (Fig 1F).

To minimize possible off-target effects of RNAi, the climbing assay was performed in an independent line of transgenic flies carrying RNAi targeting a different region of *wfs1* (Fig 1B, RNAi). Although the knockdown efficiency of this RNAi line was weaker (S1A Fig), the age-dependent decline in climbing ability was also observed in these *wfs1* knockdown flies (S1B Fig).

### Knockdown of *wfs1* in both neurons and glial cells significantly shortens the lifespan of flies and exaggerates behavioral deficits and neurodegeneration

In mammals, the WFS1 protein is mainly expressed in neurons, although expression is also observed in some glial cell types. In the context of optic atrophy, several anatomical studies reported expression of WFS1 in optic nerve astrocytes [36,37], suggesting that failure of the surrounding glial cells has detrimental effects resulting in optic nerve degeneration. In *Drosophila*, the MODENCODE database (http://www.modencode.org/) [38] indicates that *wfs1* is expressed ubiquitously in all stages of flies. However, the physiological functions of *WFS1/wfs1* in glial cells remain unclear.

**Fig 2:**
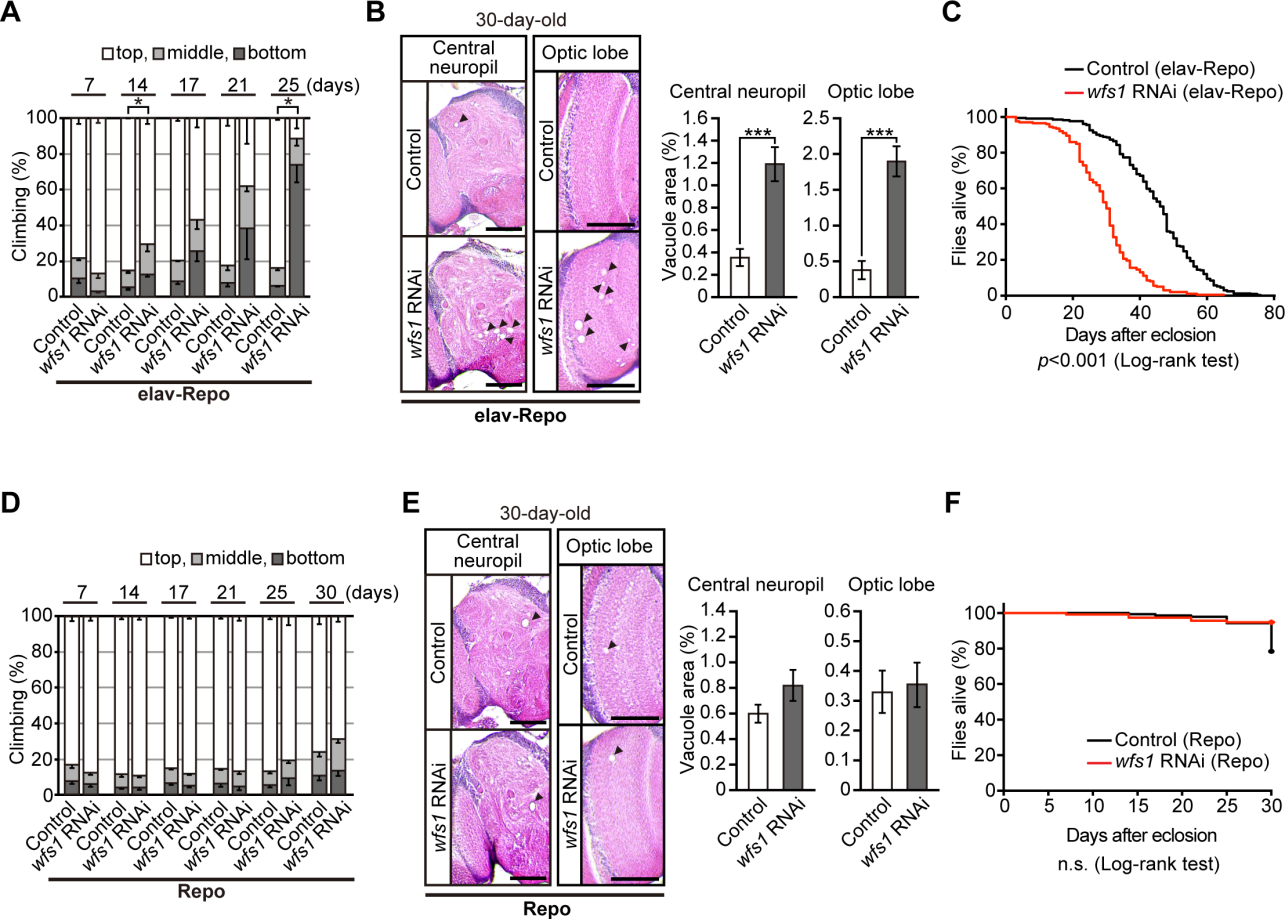
Knockdown of *wfs1* in both neurons and glial cells significantly shortens the lifespan of flies and exaggerates behavioral deficits and neurodegeneration. (**A**) Knockdown of *wfs1* in both neurons and glial cells, (**D**) but not in glial cells alone, caused age-dependent locomotor deficits as revealed by climbing assay. Average percentages of flies that climbed to the top (white), climbed to the middle (light gray), or stayed at the bottom (dark gray) of the vials. Percentages of flies that stayed at the bottom were subjected to statistical analyses. n = 3–5 independent experiments, *p < 0.05 by Student’s *t*-test. (**B**) Knockdown of *wfs1* in both neurons and glial cells, (**E**) but not in glial cells alone, caused neurodegeneration in the central neuropil or optic lobes of fly brain. Representative images show the central neuropil and optic lobe in paraffin-embedded brain section with hematoxylin and eosin (HE) staining from 30-day-old flies. Scale bars: 200 μm. Percentages of vacuole areas (indicated by arrowheads in the images) in central neuropil or optic lobes are shown. n = 14–19 hemispheres, ***p < 0.001 by Student’s *t*-test. (**C**) Knockdown of *wfs1* in both neurons and glial cells significantly shortened lifespan of flies (n = 199, *wfs1* RNAi group or 331, control group). (**F**) Knockdown of *wfs1* in glial cells did not affect lifespan up to 30 days (n = 114, *wfs1* RNAi group or 139, control group). The lifespans of flies were determined by Kaplan-Meier survival analysis with log-rank test and statistical significance was indicated in the figure. Genotypes and ages of flies are described in S1 Table.

To determine the role of wfs1 in glial cells in maintaining neuronal integrity in flies, we knocked down the expression of *wfs1* in neurons and glial cells using the *elav*-GAL4 and pan-glial *Repo*-GAL4 drivers. Knockdown of *wfs1* in neurons and glial cells exaggerated age-dependent locomotor deficits (Fig 2A) relative to those observed in flies with *wfs1* knockdown in neurons (Fig 1D) or glial cells (Fig 2D). By contrast, the severity of neurodegeneration was modestly enhanced in flies with knockdown of *wfs1* in both neurons and glial cells (Fig 2B) relative to that in flies with *wfs1* knockdown only in neurons (Fig 1E, 30 days) or glial cells (Fig 2E). Flies with knockdown of *wfs1* in both neurons and glial cells had a dramatically shorter lifespan (Fig 2C) than those with *wfs1* knockdown only in neurons (Fig 1F) or glial cells (Fig 2F). Similar effects of neuronal and glial knockdown of *wfs1* on lifespan were obtained with an independent RNAi line (S1C Fig). These results suggest that *wfs1* in neurons and glial cells act in concert to maintain neural function and integrity during aging in flies.

### Mutations in the *wfs1* gene cause behavioral deficits and neurodegeneration and shorten the lifespan of flies

We next characterized the phenotypes of *wfs1* mutant flies, a MiMIC line (Fig 1B), and PiggyBac lines (e03461 and LL07290, Fig 1B). In a line with a PiggyBac e03461 insertion located in the 5′ upstream of the *wfs1* gene (Fig 1B), qRT-PCR analysis revealed that the mRNA levels of *wfs1* were not altered (S2A Fig), and this line was excluded from further characterization. In a line with a PiggyBac LL07290 insertion located 5′ upstream of the *wfs1* gene close to the start codon (Fig 1B), the mRNA levels of *wfs1* were significantly reduced (by approximately 60%), whereas those of the adjacent gene, *Nup133*, were dramatically increased (S2B Fig). Phenotypic analysis showed age-dependent locomotor deficits (S2C Fig) and premature death (S2D Fig) in these flies.

**Fig 3:**
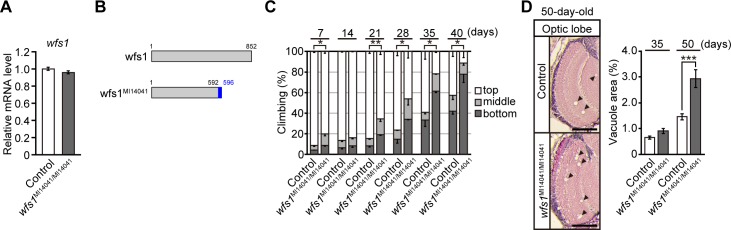
Mutations in *wfs1* gene cause behavioral deficits and neurodegeneration and shorten lifespan of flies. (**A**) mRNA levels of *wfs1* in heads of flies carrying homozygous mutations of *wfs1* with MiMIC insertion (*wfs1*^*MI14041/MI14041*^) were analyzed by qRT-PCR. n = 4. (**B**) Schematic diagrams of wild-type wfs1 protein (wfs1) and C-terminally truncated wfs1 protein by MiMIC insertion (wfs1^MI14041^). (**C**) The *wfs1* mutant flies with MiMIC insertion induced age-dependent locomotor deficits as revealed by climbing assay. Average percentages of flies that climbed to the top (white), climbed to the middle (light gray), or stayed at the bottom (dark gray) of the vials. Percentages of flies that stayed at the bottom were subjected to statistical analyses. n = 3 independent experiments, *p < 0.05 and **p < 0.01 by Student’s *t*-test. (**D**) Homozygous mutations of *wfs1* with MiMIC insertion caused age-dependent neurodegeneration in the optic lobes of fly brain. Representative images show the optic lobe in paraffin-embedded brain section with hematoxylin and eosin (HE) staining from 50-day-old flies. Scale bars: 200 μm. Percentages of vacuole areas (indicated by arrowheads in the images) in the optic lobes from 35- and 50-day-old flies were analyzed. n = 22–24 hemispheres, ***p < 0.001 by Student’s *t*-test. Genotypes and ages of flies are described in S1 Table.

In a MiMIC insertion located in the last exon of the *wfs1* gene (Fig 1B), DNA sequence analysis around the putative insertion sites showed that the wfs1 protein (852 amino acids length) ended at amino acid position 592 and was flanked by four amino acids derived from the MiMIC constructs (S3 Fig). The mRNA expression levels were not altered in these flies (Fig 3A). The MiMIC flies were therefore expected to express a C-terminally truncated version of the wfs1 protein (Fig 3B), which may act as a partial loss-of-function and/or gain-of-function mutation. These flies showed age-dependent locomotor defects (Fig 3C) and neurodegeneration (Fig 3D). Taken together, these data suggest that the neurological phenotypes induced by neuronal knockdown of *wfs1* were present in both MiMIC and PiggyBac insertion lines.

### Neuronal knockdown of *wfs1* alone does not induce ER stress, mitochondrial dysfunction, or lysosome/autophagy defects in fly brains

In mammals, the WFS1 protein mainly localizes to the ER [39] and secretory vesicles [40], and to a lesser extent to mitochondria [40,41]. To examine the cellular localization of wfs1 in *Drosophila* cells, the wfs1 protein was tagged with HA at the C-terminus and expressed in *Drosophila* S2 cells. Immunostaining with anti-HA antibody revealed that, although overlaps between wfs1 proteins and KEDL signals were weak, they partially overlapped with Concanavalin A (ConA) staining, another ER marker as well as Mitotracker signals (S4 Fig).

*WFS1* expression is induced to regulate the cellular response against ER stress in mammalian cells [8–12]. We asked whether this mechanism is evolutionally conserved in *Drosophila*. Of the three main branches of the mammalian unfolded protein response (UPR), the PERK and IRE1/XBP1 pathways are well conserved in *Drosophila* [42]; in flies, the Ire1-dependent splicing of *Xbp1* mRNA generates the active *Xbp1* isoform *Xbp1-RB*. To induce ER stress in fly brain neurons, we expressed misfolding prone amyloid-β42 peptides (Aβ42) in the ER [43]. We found that mRNA expression levels of *wfs1* along with other ER stress marker genes, including *Xbp1-RB*, *PEK* (fly ortholog of *PEK*) and the ER chaperone *Hsc70-3*, which is among the downstream targets of *Xbp1-RB*, were significantly increased in fly heads expressing Aβ42 (S5 Fig). These results suggest that fly cells are capable of inducing *wfs1* expression upon ER stress *in vivo*.

**Fig 4:**
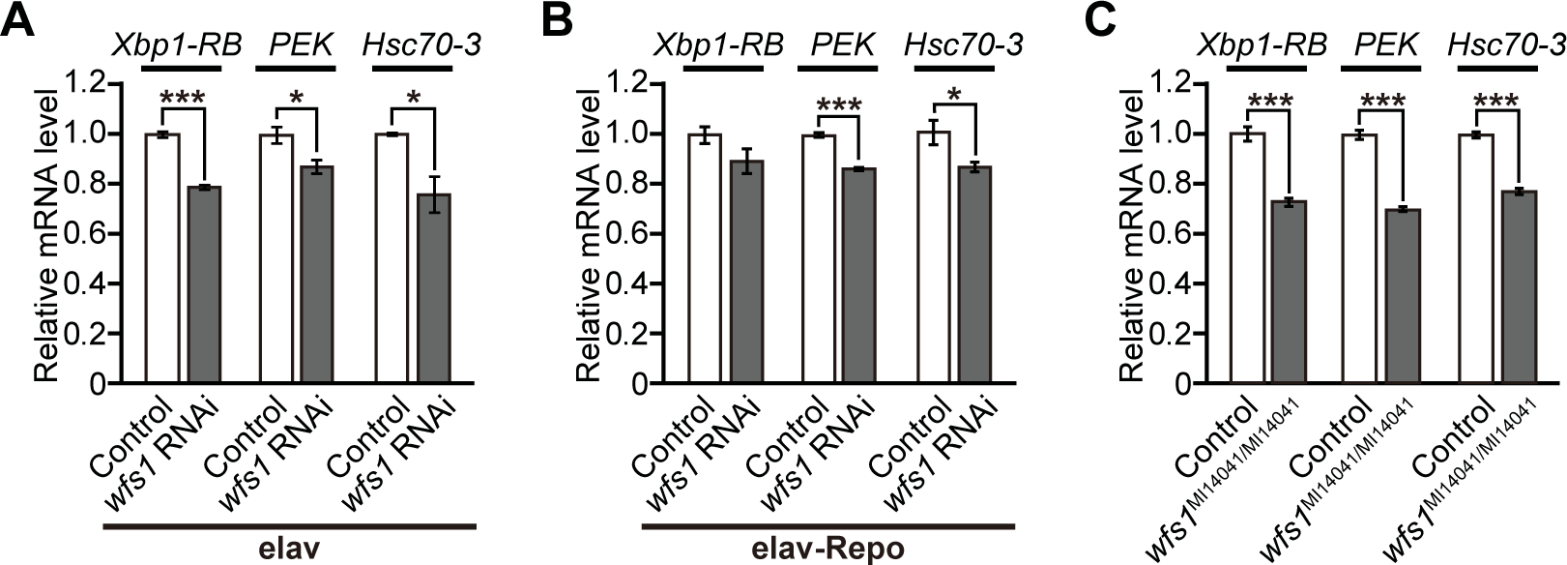
Knockdown of *wfs1* alone does not induce ER stress in the fly brains. **(A)** Neuronal (*elav*), **(B)** neuronal and glial (*elav-Repo*) knockdown of *wfs1* or **(C**) *wfs1* mutant with MiMIC insertion (*wfs1*^*MI14041/MI14041*^) did not increase mRNA levels of *Xbp1-RB*, *PEK* and *Hsc70-3* in fly brains, as determined by qRT-PCR. n = 4, *p < 0.05 and ***p < 0.001 by Student’s *t*-test. Genotypes and ages of flies are described in S1 Table.

*WFS1* deficiency leads to ER stress in pancreatic β-cell lines such as INS-1 [8,13], pancreatic β cells from *Wfs1*-deficient mice [11,16], or WS patients [10]. By contrast, activation of the UPR is not observed in non-pancreatic tissues including the brains of *Wfs1* knockout mice [11]. To determine whether knockdown of *wfs1* in the nervous system induced ER stress in fly brains, the expression of UPR-related genes was analyzed. The levels of mRNAs encoding *Xbp1-RB*, *PEK*, and *Hsc70-3* in aged fly brains were slightly lower in neuronal or neuronal and glial *wfs1* knockdown flies than in the controls (Fig 4A and 4B). Similar results were obtained in *wfs1* mutant flies (Fig 4C). These results suggest that global activation of ER stress in fly brains is not the primary cause of the neurodegenerative phenotypes induced by *wfs1* deficiency.

Because of the syndromic similarities of many clinical features between WS and mitochondrial disorders [44], the pathophysiology of WS has been attributed to mitochondrial dysfunction and mitochondrial DNA (mtDNA) defects [45–48]. A recent study using primary cultured neurons demonstrated that *WFS1* deficiency alters Ca^2+^ homeostasis in the ER and promotes mitophagy, leading to alterations in mitochondrial distribution and dynamics, reductions in ATP levels, and retardation of neuronal development [49]. These reports suggest that mitochondrial defects also contribute to neurodegeneration caused by *WFS1* deficiency.

We investigated whether *wfs1* deficiency would induce severe mitochondrial dysfunction in fly brains. The results showed that total ATP content did not differ significantly between control and *wfs1* knockdown brains (S6A Fig). In addition, *wfs1* deficiency did not dramatically alter the mRNA expression levels of genes involved in mitochondrial fission and fusion, including *Marf*, *Opa1*, *Fis1*, and *Drp1* (S6B Fig), or the levels of mitochondrial-resident proteins, including VDAC1, NDUFS3, and MnSOD (S6C Fig). To determine whether neuronal knockdown of *wfs1* affected mitochondrial distribution in fly brains, the mitochondrial marker mito-GFP was expressed in the mushroom body structures of the fly brain, where its distribution in the cell body, dendrites, and axons of mushroom body neurons could be easily distinguished and quantified (S6D Fig). The results showed that *wfs1* deficiency did not significantly disrupt mito-GFP distribution in mushroom body neurons (S6E Fig). Finally, assessment of possible genetic interactions between *wfs1* and *Opa1* showed that mutation of *Opa1* did not exacerbate the age-dependent neuronal dysfunction caused by *wfs1* deficiency (S6F Fig).

To determine whether neuronal knockdown of *wfs1* affects autophagy in aged fly brains, the levels of the ref(2)P protein and the Atg8-II/Atg8-I ratio were measured. If autophagy were compromised, ref(2)P levels and the Atg8-II/Atg8-I ratio would increase. There were no significant changes in the levels of ref(2)P or the Atg8-II/Atg8-I ratio in *wfs1*-knockdown flies, suggesting that autophagy efflux is not compromised by *wfs1* knockdown (S7 Fig).

Taken together, these results indicated that *wfs1* deficiency did not induce severe defects in mitochondrial and autophagy functions in fly brains.

### Oxidative stress induces *wfs1* expression, and neuronal knockdown of *wfs1* increases vulnerability to oxidative stress

**Fig 5:**
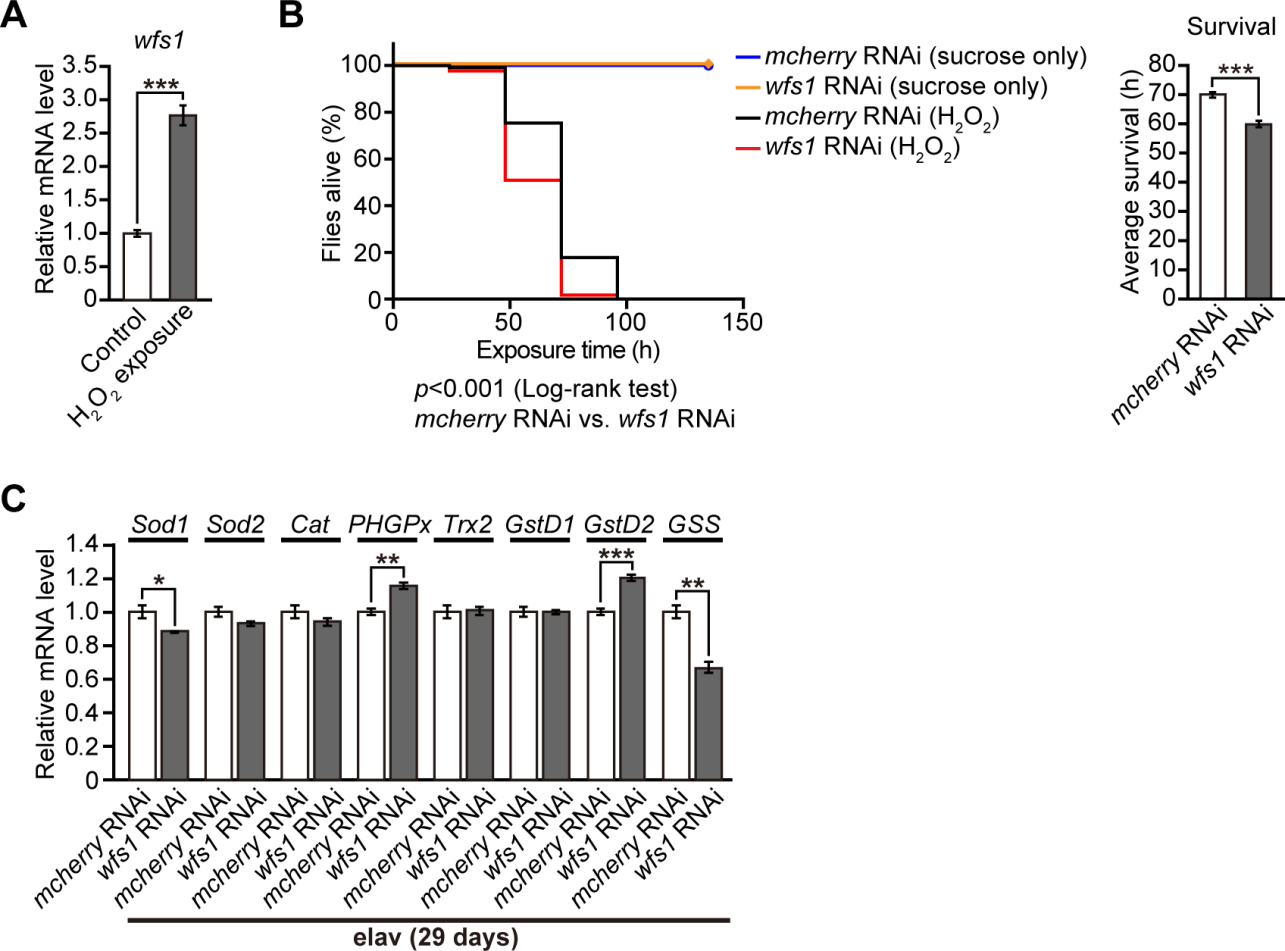
Oxidative stress induces *wfs1* expression, and neuronal knockdown of *wfs1* increases vulnerability to oxidative stress. (**A**) mRNA levels of *wfs1* were induced by hydrogen peroxide (H_2_O_2_) exposure in fly brains, as determined by qRT-PCR. n = 4, ***p < 0.001 by Student’s *t*-test. (**B**) Neuronal knockdown of *wfs1* significantly reduced survival time after H_2_O_2_ exposure compared with that of flies with *mcherry* RNAi. The survival rates of flies with H_2_O_2_ exposure were determined by Kaplan-Meier survival analysis with log-rank test and statistical significance was indicated in the figure (left panel) (n = 29, *mcherry* RNAi with 10% sucrose only, n = 26, *wfs1* RNAi with 10% sucrose only, n = 236, *mcherry* RNAi with H_2_O_2_ exposure, n = 171, *wfs1* RNAi with H_2_O_2_ exposure). Average survival time of *wfs1* knockdown flies with H_2_O_2_ exposure was significantly reduced (right panel). ***p <0.001 by Student’s *t*-test. (**C**) The mRNA levels of genes related to oxidative stress responses in 29-day-old fly brains with *wfs1* RNAi or *mcherry* RNAi were determined by qRT-PCR. n = 4, *p < 0.05, **p < 0.01 and ***p < 0.001 by Student’s *t*-test. Genotypes and ages of flies are described in S1 Table.

The results described in the past sections suggested that *wfs1* deficiency by itself did not cause severe cellular dysfunction directly leading to neurodegeneration in fly brains. Since age-dependent neurodegeneration often involves oxidative stress, we first examined whether *wfs1* plays a role in the oxidative stress response in flies. Exposure of flies to hydrogen peroxide (H_2_O_2_) significantly increased the mRNA levels of *wfs1* in fly brains (Fig 5A), and neuronal knockdown of *wfs1* significantly reduced the survival of flies under oxidative stress conditions (Fig 5B), suggesting that *wfs1* plays a protective role against oxidative stress. We next examined whether *wfs1* deficiency by itself was sufficient to induce an oxidative stress response under normal conditions. Despite small increases or decreases in the mRNA expression levels of oxidative stress-related genes, neuronal knockdown of *wfs1* did not induce a severe oxidative stress response in fly brains (Fig 5C). Taken together, these results suggest that *wfs1* deficiency in neurons increases the vulnerability to oxidative stress in flies.

### Heterozygous mutation in *Eaat1*, a glial high-affinity glutamate transporter, significantly worsens the behavioral deficits caused by *wfs1* deficiency

The brain damage that occurs after ischemic stroke and in some neurodegenerative disorders is partially due to excessive quantities of extracellular glutamate and the concomitant overactivation of glutamate receptors, leading to excitotoxicity [50,51]. Extracellular glutamate levels are tightly regulated to enable the precise control of neurotransmission at glutamatergic synapses while preventing neuronal cell death. WS patients frequently develop diverse neurological complications including epilepsy [19]. In a rat model of epilepsy induced by kainic acid toxicity, *Wfs1* mRNA levels are markedly reduced in the hippocampal CA3 region [52]. *Wfs1* mRNA levels are elevated in aged rodent brains [34], and this increase is reversed by treatment with riluzole, a drug that inhibits glutamate release. Taken together, these findings suggest a potential link between aging, neuronal excitability, and *wfs1* function in the brain.

**Fig 6:**
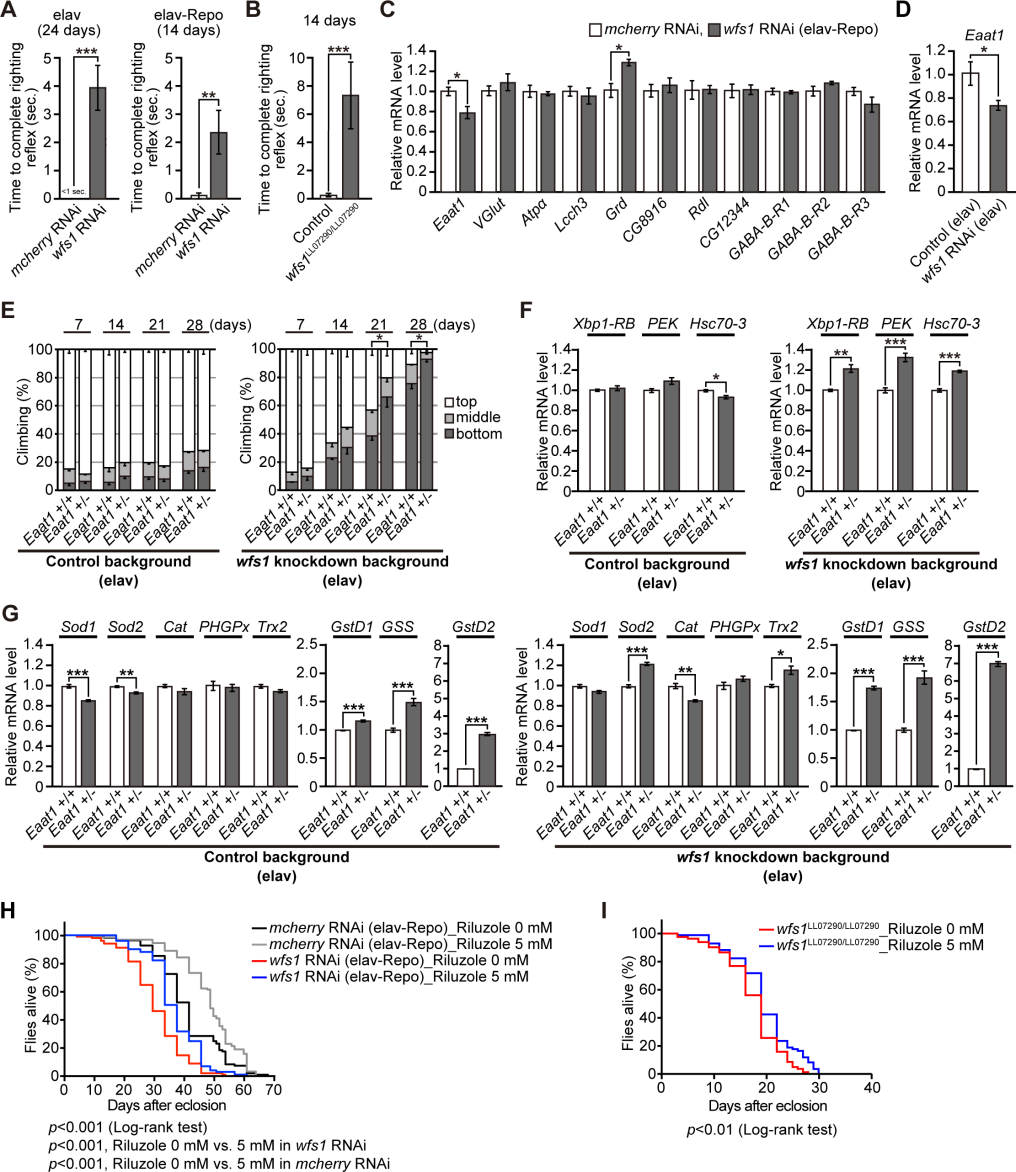
Heterozygous mutation in *Eaat1*, a glial high-affinity glutamate transporter, significantly worsens behavioral deficits caused by *wfs1* deficiency. (**A**) Flies with neuronal (*elav*), neuronal and glial (*elav-Repo*) knockdown of *wfs1* or **(B**) *wfs1* mutant with PiggyBac insertion (*wfs1*^*LL07290/LL07290*^) required longer time to complete righting reflex than control flies. n = 10–26, **p < 0.01 and ***p < 0.001 by Mann-Whitney *U*-test. (**C**) The mRNA levels of genes related to synaptic activities with focus on GABAergic as well as glutamatergic systems in fly brains with neuronal and glial knockdown of *wfs1* were determined by qRT-PCR. n = 4, *p < 0.05 by Student’s *t*-test. (**D**) The mRNA level of *Eaat1* in fly brains with neuronal knockdown of *wfs1* was determined by qRT-PCR. n = 4, *p < 0.05 by Student’s *t*-test. (**E**) Heterozygous mutation in *Eaat1* did not induce locomotor defects in aged flies (left panel). In the *wfs1* knockdown background, heterozygous mutation in *Eaat1* caused locomotor deficits (right panel) as revealed by climbing assay. Average percentages of flies that climbed to the top (white), climbed to the middle (light gray), or stayed at the bottom (dark gray) of the vials. Percentage of flies that stayed at the bottom were subjected to statistical analyses. n = 4 independent experiments. n = 5, *p < 0.05 by Student’s *t*-test. (**F**) A heterozygous mutation in *Eaat1* induced ER stress responses in *wfs1* knockdown background, but not in control background. The mRNA levels of *Xbp1-RB*, *PEK* and *Hsc70-3* in fly brains were determined by qRT-PCR. n = 4, *p < 0.05, **p < 0.01 and ***p < 0.001 by Student’s *t*-test. (**G**) mRNA levels of genes related to oxidative stress responses in fly heads carrying a heterozygous mutation in *Eaat1* were prominently elevated in *wfs1* knockdown background compared to control background, as determined by qRT-PCR. n = 4, *p < 0.05, **p < 0.01 and ***p < 0.001 by Student’s *t*-test. (**H**-**I**) Riluzole increased the survival time in flies with neuronal and glial knockdown of *wfs1* (**H**) and in flies carrying homozygous mutations of *wfs1* with PiggyBac insertion (*wfs1*^*LL07290/LL07290*^) (**I**). The survival rates were determined by Kaplan-Meier survival analysis with log-rank test, and Holm-Sidak method was used for multiple comparison analysis (n = 138, *mcherry* RNAi with Riluzole 0 mM, n = 128, *mcherry* RNAi with Riluzole 5 mM, n = 102, *wfs1* RNAi with Riluzole 0 mM, n = 101, *wfs1* RNAi with Riluzole 5 mM, n = 82, *wfs1*^LL07290/LL07290^ with Riluzole 0 mM, n = 85, *wfs1*^LL07290/LL07290^ with Riluzole 5 mM). The statistical significance was indicated in the figure. Genotypes and ages of flies are described in S1 Table.

Flies with knockdown of *wfs1* in the nervous system exhibited a high frequency of supine behavior and a longer time to right from this position when the flies were tapped down to the bottom of the vials. The righting reflex assay [53] showed that flies with neuronal or both neuronal and glial knockdown of *wfs1* (Fig 6A) and *wfs1* mutant flies (Fig 6B) required a significantly longer time to right from the supine position. Since the supine behavior is induced by altered GABAergic activity [53], the mRNA expression levels of genes related to synaptic activities were examined, focusing on GABAergic and glutamatergic systems. The mRNA levels of seven out of eight genes encoding GABA receptors, except for *Grd*, a fly ortholog of GABA-A receptor α subunit, were not altered by neuronal and glial knockdown of *wfs1*, whereas the mRNA levels of *Eaat1*, a single high-affinity glutamate transporter expressed in glial cells, were significantly decreased in *wfs1* knockdown flies (Fig 6C). We also found that the neuronal knockdown of *wfs1* was sufficient to reduce mRNA expression levels of *Eaat1* in fly heads (Fig 6D). These results suggest that neuronal knockdown of *wfs1* down-regulates *Eaat1* levels in glial cells in a cell non-autonomous manner and this may cause an imbalance in neural activity in flies.

Next, we investigated whether *wfs1* deficiency increases the susceptibility of flies to neuronal dysfunction caused by altered neuronal excitability. In *Drosophila*, loss of *Eaat1* causes age-dependent locomotor defects [54,55]. We found that the heterozygous *Eaat1* mutant alone did not cause locomotor defects up to 28 days of age (Fig 6E, left). By contrast, in the *wfs1* knockdown background, the heterozygous *Eaat1* mutation caused locomotor defects starting at 21 days (Fig 6E, right). These results suggest that *wfs1* deficiency renders neurons vulnerable to neuronal dysfunction caused by altered neuronal excitability.

To investigate the mechanisms underlying the genetic interaction between *Eaat1* and *wfs1*, we first examined whether the UPR was induced in flies with a combination of *Eaat1* heterozygous mutations and neuronal knockdown of *wfs1*. *Eaat1* heterozygous mutations significantly upregulated the mRNA expression of UPR marker genes in the *wfs1* knockdown background, but not in the wild-type background in fly heads (Fig 6F). Since neuronal knockdown of *wfs1* alone did not induce the expression of UPR marker genes (Fig 4A), these results suggest that knockdown of *wfs1* renders brain cells (neurons and/or glial cells) vulnerable to ER stress associated with reduced Eaat1 function.

Oxidative stress is induced by impaired Eaat1 function in flies [54]. We therefore examined the effect of increased oxidative stress on the genetic interaction between *Eaat1* and *wfs1*. qRT-PCR analysis showed that a heterozygous mutation of *Eaat1* alone slightly upregulated the mRNA expression of three of eight oxidative stress-related genes in the wild-type background (Fig 6G, left), and this effect was enhanced in flies with neuronal knockdown of *wfs1*, in which five of eight genes were significantly upregulated at the mRNA level (Fig 6G, right). These results suggest that knockdown of *wfs1* imposes oxidative stress to the cells under reduced Eaat1 function, which may contribute to the increased behavioral deficits in these flies.

We further examined whether correcting the imbalance of neural activity could suppress neuronal dysfunction in *wfs1* knockdown flies. Since *Eaat1* expression was decreased (Fig 6C and 6D) and the loss of one copy of *Eaat1* worsened the behavioral deficits in *wfs1* knockdown flies (Fig 6E), we treated flies with riluzole, which inhibits glutamate release and reduces extracellular glutamate levels, thereby suppressing excitotoxicity [54]. Although administration of riluzole did not suppress age-dependent locomotor defects (S8A Fig), it significantly increased the survival of *wfs1* knockdown and mutant flies (Fig 6H and 6I). Interestingly, administration of riluzole also increased the lifespan of control flies (Fig 6H, *mcherry* RNAi), suggesting that riluzole treatment extends the lifespan of *wfs1* knockdown flies by suppressing general age-related stresses. Such protective effects were not observed in flies treated with the anticholinergic agent orphenadrine (S8B and S8C Fig).

Taken together, these results suggest that behavioral deficits in *wfs1* knockdown flies partly involve glutamatergic imbalance, and *wfs1* deficiency increases the susceptibility to neuronal dysfunction caused by altered neuronal excitability in flies.

### Neuronal knockdown of *wfs1* increases susceptibility to axon degeneration caused by overexpression of human tau

**Fig 7:**
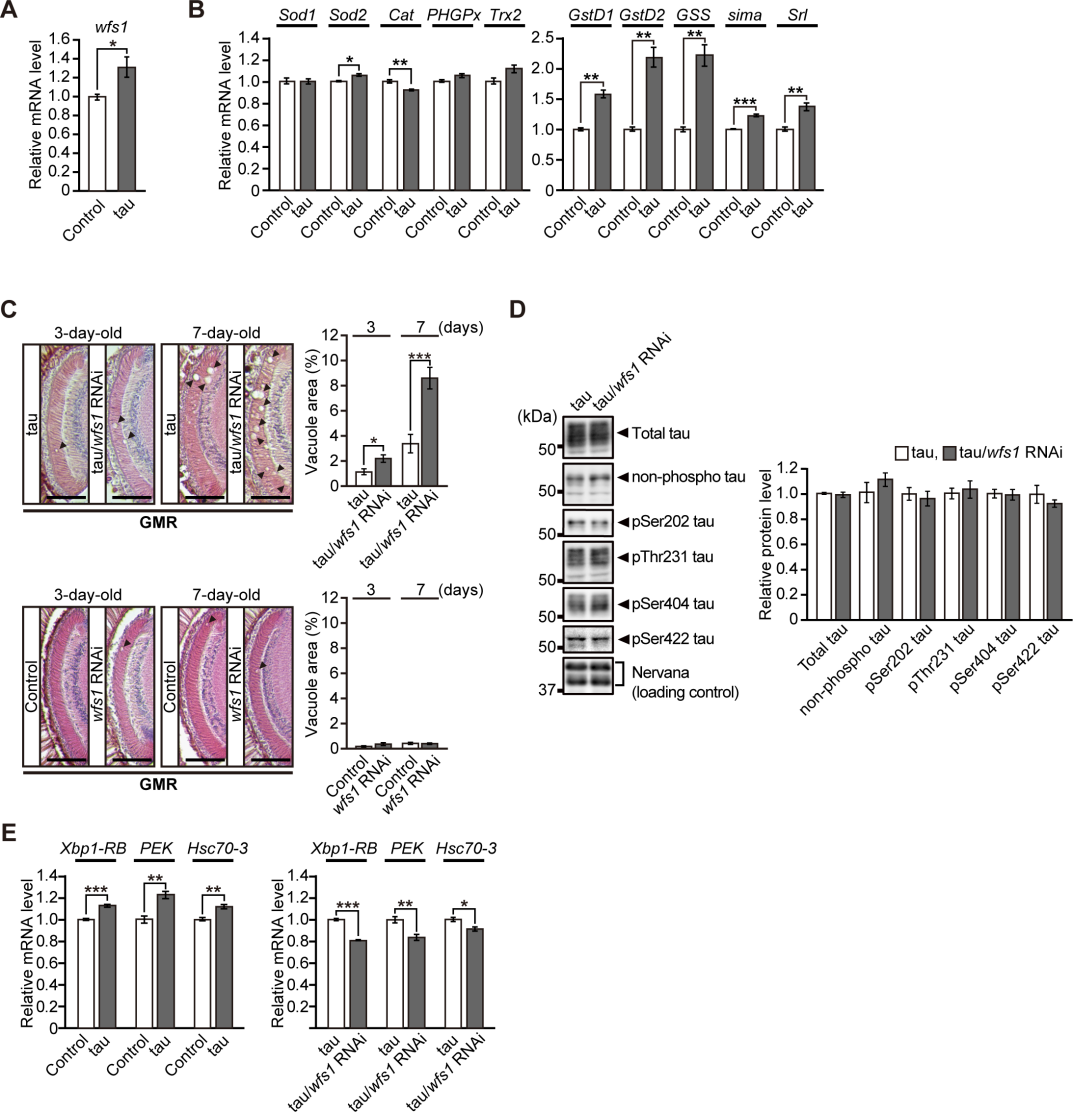
Neuronal knockdown of *wfs1* increases susceptibility to axon degeneration caused by overexpression of human tau. (**A**) mRNA levels of *wfs1* were induced by ectopic expression of human microtubule associated protein tau in fly eyes, as determined by qRT-PCR. n = 4, *p < 0.05 by Student’s *t*-test. (**B**) The mRNA levels of genes related to oxidative stress responses in flies expressing human tau were determined by qRT-PCR. n = 4, *p < 0.05, **p < 0.01 and ***p < 0.001 by Student’s *t*-test. (**C**) Neuronal knockdown of *wfs1* exaggerated axon degeneration in the lamina caused by ectopic overexpression of human tau (upper panels). Neuronal knockdown of *wfs1* alone did not cause axon degeneration in the lamina (lower panels). Representative images show the lamina in paraffin-embedded head section with hematoxylin and eosin (HE) staining from 3- and 7-day-old flies. Scale bars: 200 μm. Percentages of vacuole areas in the lamina (indicated by arrowheads in the images) are shown. n = 9–12 hemispheres, *p < 0.05 and ***p < 0.001 by Student’s *t*-test. (**D**) Neuronal knockdown of *wfs1* did not alter either the total protein levels or phosphorylation levels of tau. Fly heads expressing tau alone (tau) or co-expressing tau and RNAi transgene for *wfs1* (tau/*wfs1* RNAi) were subjected to western blotting with anti-tau or anti-phospho tau antibodies. Nervana, a fly ortholog of ATPase Na+/K+ transporting subunit β 1, was used as the loading control. n = 3–4, no significant difference. (**E**) Overexpression of human tau in the lamina induced ER stress responses compared with control (left panel). Neuronal knockdown of *wfs1* did not further increase ER stress responses caused by tau expression (right panel). The mRNA levels of *Xbp1-RB*, *PEK* and *Hsc70-3* in fly heads were determined by qRT-PCR. n = 4, *p < 0.05, **p < 0.01 and ***p < 0.001 by Student’s *t*-test. Genotypes and ages of flies are described in S1 Table.

Post-mortem studies show widespread axonal pathology in WS patients [56–58]. To determine whether *wfs1* knockdown exacerbated axon degeneration, we used transgenic *Drosophila* expressing the Alzheimer’s disease-related human tau protein under the control of the pan-retinal GMR-GAL4 driver. Ectopic expression of human tau causes age-dependent and progressive degeneration in the lamina, the first synaptic neuropil of the optic lobe, which contains photoreceptor axons [59]. We found that *wfs1* expression was induced by tau expression (Fig 7A) concomitant with a significant upregulation of the mRNA expression of oxidative stress-related genes (Fig 7B). These results suggest that *wfs1* plays a protective role against tau-mediated neurodegeneration. Knockdown of *wfs1* significantly increased axon degeneration caused by tau in the lamina at 3 and 7 days of age (Fig 7C, upper panels), whereas knockdown of *wfs1* alone did not cause axon degeneration in the lamina at these ages (Fig 7C, lower panels). Knockdown of *wfs1* did not alter tau total protein or phosphorylation levels (Fig 7D). We further examined whether overexpression of *wfs1* could suppress tau-mediated neurodegeneration. Although there was a trend that *wfs1* expression slightly suppressed retinal degeneration induced by tau, they did not reach statistical significance (S9 Fig).

To explore the possible involvement of ER stress in the enhancement of neurodegeneration, we examined whether UPR gene expression was induced in flies with a combination of tau expression and *wfs1* knockdown under the control of the GMR-GAL4 driver. Expression of tau alone in retinal cells slightly upregulated the mRNA expression of UPR marker genes (Fig 7E, left). However, the combination of tau expression and *wfs1* knockdown did not further increase, but rather slightly decreased the expression levels of UPR marker genes (Fig 7E, right), suggesting that an increased UPR is not the primary mechanism underlying enhanced tau toxicity.

Taken together, these results suggest that *wfs1* knockdown increases the vulnerability of neurons to axon degeneration induced by tau.

## Discussion

Most WS patients die prematurely in association with severe neurological disabilities as a result of brain atrophy [1,24,25]; however, the mechanisms underlying neurodegeneration caused by *WFS1* deficiency remain elusive. In this study, we investigated the effects of knockdown of *wfs1*, a fly homolog of *WFS1*, in the nervous system on neuronal integrity during aging. The results demonstrated that *wfs1* expression was induced upon aging, and neuronal knockdown of *wfs1* caused age-dependent behavioral deficits and neurodegeneration (Fig 1 and S1 Fig).

The process by which *wfs1* deficiency induced behavioral deficits and neurodegeneration in our *Drosophila* model remained unclear. In pancreatic β cells, *WFS1* deficiency causes chronic ER stress and subsequent cell death through the dysregulation of intracellular Ca^2+^ levels [14,15] and disruption of ER homeostasis [8,9,11,16]. In addition, a recent report using primary cultured neurons demonstrated that *WFS1* deficiency alters Ca^2+^ homeostasis in the ER and promotes mitophagy, leading to alterations in mitochondrial distribution and dynamics [49]. We found that knockdown of *wfs1* in the nervous system did not induce ER stress (Fig 4) or mitochondrial dysfunction (S6 Fig) even in aged fly brains. Moreover, autophagy flux was not significantly affected in these flies (S7 Fig). These results suggest that reductions in *wfs1* function alone do not severely disrupt cellular functions, which directly causes neurodegeneration. Rather, *wfs1* may play a protective role against various stressors associated with aging, and its deficiency increased the susceptibility to neurodegeneration in aged flies.

In support of this hypothesis, *wfs1* expression was induced by aging and stressors (Figs 1, 5 and 7 and S5 Fig), and neuronal knockdown of *wfs1* rendered neurons vulnerable to oxidative stress-, altered neuronal excitability- or Alzheimer’s associated tauopathy-induced behavioral deficits and neurodegeneration (Figs 5, 6 and 7). Moreover, although knockdown of *wfs1* in neurons did not induce obvious stress responses (Figs 4 and 5C), *wfs1* deficiency significantly augmented oxidative stress and ER stress responses under stressed conditions (Fig 6F and 6G). These results suggest that *wfs1* deficiency modifies the onset, progression and severity of neurological and neurodegenerative phenotypes caused by a combination of complex environmental, genetic, and age-associated factors.

In this study, knockdown of *wfs1* in neurons and glial cells significantly exaggerated behavioral deficits and premature death (Fig 2), suggesting that neuronal and glial *wfs1* work in concert to maintain neuronal integrity in flies. The mRNA expression levels of *Eaat1* and *Grd*, which are fly orthologs of glial glutamate transporters (*SLC1A3*) and GABA-A receptor α subunit (*GABRA6*), respectively, were significantly altered in these fly brains (Fig 6C). Moreover, loss of one copy of *Eaat1* increased locomotor deficits induced by *wfs1* deficiency (Fig 6E), whereas feeding of riluzole, which is thought to inhibit glutamate release as well as GABA uptake, significantly suppressed premature death phenotypes in these flies (Fig 6H and 6I). These results suggest that stresses caused by altered neuronal excitability, possibly due to an imbalance between glutamatergic and GABAergic tones, may underlie the observed behavioral deficits in flies with *wfs1* deficiency.

Recent reports suggest that the neurological manifestations of *WFS1* deficiency are associated with endocrine problems [3,20]. In addition to the ER, the WFS1 protein localizes to secretory granules and regulates intragranular acidification, proinsulin processing, and exocytosis in pancreatic β cells [40], in part by interacting with the V1A subunit of H+ V-ATPase [60]. Consistent with this, vasopressin processing is defective in some hypothalamic nuclei in the brain of WS patients [61], and the processing and secretion of growth and trophic factors is impaired in *Wfs1-*deficient mice [31]. We examined whether knockdown of *wfs1* in neurons and glial cells alters the levels of a fly ortholog of Mesencephalic Astrocyte-derived Neurotrophic Factor (MANF) [62] in fly brains. Western blot analyses revealed that the level of MANF was not significantly altered in fly brains (S10 Fig). Further analysis is required to clarify the mechanism by which *wfs1* functions in neurons and glial cells to maintain neuronal function and integrity in fly brains.

The *wfs1* mutant and RNAi lines used in this study did not completely abolish *wfs1* expression and function in flies. This indicates that some of the phenotypes induced by knockdown of *wfs1* may be different from those caused by complete null mutation of *wfs1*. Nevertheless, this study demonstrated that *wfs1* deficiency in the nervous system is sufficient to render neurons vulnerable to age-associated neurodegeneration in *Drosophila*. Our results also highlight the importance of the functions of *wfs1* in both neuronal and glial cells. Moreover, our data revealed a novel genetic interaction between *wfs1* and *Eaat1*, and suggest that altered neural activity and vulnerability to oxidative stress underlie neurological phenotypes in *wfs1* deficient flies.

In conclusion, this study provides insight into the molecular mechanisms underlying neurodegeneration in WS and furthers our understanding of potential disease-modifying factors that determine susceptibility and resilience to age-associated neurodegenerative diseases such as Alzheimer’s disease.

## Materials and methods

### *Drosophila* genetics

Flies were maintained in standard cornmeal media at 25°C. Transgenic fly lines carrying *UAS-Luciferase RNAi*, *UAS-human tau* and *UAS-Aβ42* were previously described [43,63]. *Eaat1*^*SM2*^ and a precise excision line *Eaat1*^*PE*^ (used as a control line for *Eaat1*^*SM2*^) are kind gifts from Dr. D. J. van Meyel (McGill University) [55]. The *elav-GAL4* (#458), *Repo-GAL4* (#7415), *GMR-GAL4* (#1104), *UAS-wfs1 RNAi*^*TRiP*^ (#53330), *UAS-mcherry RNAi* (#35785), *wfs1*^*e03461*^ (#18157), *wfs1*^*MI14041*^ (#59250), *UAS-wfs1* (#8356), *UAS-wfs1* (#8357), *UAS-mitoGFP* (#8442) and *UAS-Luciferase* (#35788), *Opa1*^*S3475*^ (#12188) were obtained from the Bloomington Stock Center. *UAS-wfs1 RNAi*^*GD*^ (#42664) was obtained from the Vienna *Drosophila* RNAi Center. *wfs1*^*LL07290*^ (#142011) was obtained from Kyoto Stock Center. Genotypes and ages of all flies used in this study are provided in S1 Table. Experiments were performed using age-matched male or female flies.

### Climbing assay

The forced climbing assay was performed as previously described [63,64]. Approximately 25 male flies were placed in an empty plastic vial. The vial was then gently tapped to knock all of the flies to the bottom. The numbers of flies in the top, middle, or bottom thirds of the vial were scored after 10 seconds. The percentages of flies that stayed at the bottom were subjected to statistical analyses.

### Histological analysis

Preparation of paraffin sections, hematoxylin and eosin staining, and analysis of neurodegeneration were performed as described previously [59]. Heads of male or female flies were fixed in 4% paraformaldehyde for 24 h at 4°C and embedded in paraffin. Serial sections (6μm thickness) through the entire heads were prepared, stained with hematoxylin and eosin (Sigma-Aldrich), and examined by bright-field microscopy. Images of the sections were captured with AxioCam 105 color (Carl Zeiss). For quantification, we focused on analyzing sections covering the optic lobe, central neuropil, or lamina regions. We selected a section with the most severe neurodegeneration in each individual fly and the area of vacuoles was measured using Image J (NIH). Images of the external eye structures were captured with OLYMPUS DP26 CCD Camera (OLYMPUS), and the external eye areas were measured by ImageJ.

### Lifespan analysis

Lifespan analysis was performed as described previously [63,64]. Food vials containing 25 male flies were placed on their sides at 25°C under conditions of 70% humidity and a 12:12-h light:dark cycle. Food vials were changed every 3–4 days, and the number of dead flies was counted each time. At least four vials for each genotype were prepared.

### Hydrogen peroxide exposure assays

Flies were starved with 2% agar medium for 2 hrs before hydrogen peroxide (H_2_O_2_) treatment. Round filter paper was placed on the bottom of empty vial and 120 μl of 5% H_2_O_2_ (Wako Pure Chemical) in 10% sucrose (Nacalai Tesque) solution was added to the filter paper. The dead flies were scored every 24 hrs. At least 85 male flies (11-day-old) per genotype were used to perform this experiment. For control experiment, 25–30 male flies (15-day-old) per genotype were administrated with 120 μl of 10% sucrose solution.

### Righting reflex assay

We slightly modified the protocol described in the previous study [53]. A single male fly was placed in a 1.5 ml micro test tube and was subjected to a brief mechanical shock by vortexing for 5 seconds. The fly was tapped into a supine position and the time required to right itself was measured. At least ten flies were used to calculate the mean value and statistical significance was determined as described below.

### Drug feeding

Flies were fed with the food containing 0.2, 1, or 5 mM riluzole (Tokyo Chemical Industry), 10, 100, or 1000 μM orphenadrine (Tokyo Chemical Industry) or vehicle (final concentration 1% ethanol for riluzole or final concentration 1% water for orphenadrine) from the day after eclosion. These food vials were changed every 3–4 days.

### Western blotting

Western blotting was performed as described previously [59]. Ten fly heads for each genotype were homogenized in Tris-Glycine SDS sample buffer, and the same amount of the lysate was loaded to each lane of 10%, 12.5% or 15% Tris-Glycine gels and transferred to nitrocellulose membrane. The membranes were blocked with 5% nonfat dry milk, blotted with the antibodies described below, incubated with appropriate secondary antibody and developed using ECL Prime Western Blotting Detection Reagent (GE Healthcare Life Sciences). The membranes were also probed with anti-tubulin, and used as the loading control for other blots in each experiment. Anti-tubulin (Sigma-Aldrich), anti-tau (Merck Millipore), anti-non phospho tau (Merck Millipore), anti-pSer202 tau (Thermo Fisher Scientific), anti-pThr231 tau (Thermo Fisher Scientific), anti-pSer404 tau (Abcam), anti-pSer422 tau (Abcam), anti-Nervana (Developmental Studies Hybridoma Bank), anti-VDAC1 (Abcam), anti-NDUFS3 (Abcam), anti-MnSOD (Enzo Life Sciences), anti-Atg8 (Merck Millipore) and anti-ref(2)P (Abcam) antibodies were purchased. Anti-MANF is a kind gift from Dr. T. I. Heino (University of Helsinki). Imaging was performed with ImageQuant LAS 4000 (GE Healthcare Life Sciences), and the signal intensity was quantified using Image J (NIH).

### RNA extraction and quantitative real time PCR analysis

Quantitative real time PCR analysis was performed as described previously [59]. More than 25 flies for each genotype were collected and frozen. Heads were mechanically isolated, and total RNA was extracted using TRIzol Reagent (Thermo Fisher Scientific) according to the manufacturer’s protocol with an additional centrifugation step (16,000 × *g* for 10 min) to remove cuticle membranes prior to the addition of chloroform. Total RNA was reverse-transcribed using PrimeScript RT-PCR kit (TaKaRa Bio), and the resulting cDNA was used as a template for PCR on a CFX96 real time PCR detection system (Bio-Rad Laboratories). The average threshold cycle value was calculated from at least three replicates per sample. Expression of genes of interest was standardized relative to GAPDH1. Primer sequences used in this study were described below;

*wfs1* (*Drosophila*) 167 bp (for the Bloomington line 53330)

Forward; 5’-CCGAGACGAATACTCTGCCC-3’

Reverse; 5’-ATTGGACACCCGCGAGATTT-3’

*wfs1* (*Drosophila*) 189 bp (for the VDRC line 42664)

Forward; 5’-CTTCTGGTGCCTGTCTTCGT-3’

Reverse; 5’-CAACAAGCACGCTGTTGTGA-3’

*Xbp1-RB* (*Drosophila*) 238 bp

Forward; 5’-CAACCTTGGATCTGCCGCAGGG-3’

Reverse; 5’-CGCTCCAGCGCCTGTTTCCAG-3’

*PEK* (*Drosophila*) 241 bp

Forward; 5’-CTGCGCAGTCTTCGGGACGG-3’

Reverse; 5’-AGCTGCTGAAGGTGCGGCTG-3’

*Hsc70-3* (*Drosophila*) 95 bp

Forward; 5’-TCCCGATGCCGATCCCGAGG-3’

Reverse; 5’-CGCCAGCACCCTGGTACAGC-3’

*Sod1* (*Drosophila*) 160 bp

Forward; 5’-GTTCGGTGACAACACCAATG-3’

Reverse; 5’-GGAGTCGGTGATGTTGACCT-3’

*Sod2* (*Drosophila*) 189 bp

Forward; 5’-GCTTCAACAAGAAGTCGGGC-3’

Reverse; 5’-AGATGTCATCCCAGTTGGCG-3’

*Cat* (*Drosophila*) 287 bp

Forward; 5’-TACGAGCAGGCCAAGAAGTT-3’

Reverse; 5’-ACCTTGTACGGGCAGTTCAC-3’

*PHGPx* (*Drosophila*) 105 bp

Forward; 5’-ACCTAAAGGCCAAGCAGACC-3’

Reverse; 5’-TCGGGGCATATCGGTTGATG-3’

*Trx2* (*Drosophila*) 282 bp

Forward; 5’-ATCTCGATGGACAGCTGACC-3’

Reverse; 5’-CCTTGATGACATCCTCCAGAC-3’

*GstD1* (*Drosophila*) 117 bp

Forward; 5’-TGGGAACGCTGTACCAGAG-3’

Reverse; 5’-AGGTGTTCAGGAACTCGAAGG-3’

*GstD2* (*Drosophila*) 66 bp

Forward; 5’-GCCGCACGGTCATCATG-3’

Reverse; 5’-TGGTGTTCAGTAGCTTCTTGTTCAG-3’

*GSS* (*Drosophila*) 223 bp

Forward; 5’-TGGGACCAGCAAGTAAAACC-3’

Reverse; 5’-TCGCGAATGTAGAACTCGTG-3’

*Eaat1* (*Drosophila*) 188 bp

Forward; 5’-TGCTCTGTTCATCGCCCAAT-3’

Reverse; 5’-CGACGGCTATGATGAGGGAC-3’

*VGlut* (*Drosophila*) 164 bp

Forward; 5’-TTCATCGCCTCCAAGTTCCC-3’

Reverse; 5’-GCTGGATAGGTAACGCCCTC-3’

*Atpα* (*Drosophila*) 190 bp

Forward; 5’-ACATGGTGCCAGCCATTTCA-3’

Reverse; 5’-AAGCCGTTCTCAGCCATGAT-3’

*Lcch3* (*Drosophila*) 183 bp

Forward; 5’-CCGAGACGTGTTCAACGACA-3’

Reverse; 5’-GGCTATGTCCGGTGCCATAA-3’

*Grd* (*Drosophila*) 194 bp

Forward; 5’-TTTGGCTACACAACGTCGGA-3’

Reverse; 5’-GGTCGTGGTGGATCCTTGTT-3’

*CG8916* (*Drosophila*) 182 bp

Forward; 5’-TTGAGTCCAAGAGCGGTGTC-3’

Reverse; 5’-CGTTTGGGTGGTTCTCTCCA-3’

*Rdl* (*Drosophila*) 186 bp

Forward; 5’-TGGCTCAATCGCAATGCAAC-3’

Reverse; 5’-GACCGTGGCGTATTCCAGTA-3’

*CG12344* (*Drosophila*) 178 bp

Forward; 5’-CGAGAGCTTCTCGTCGAACA-3’

Reverse; 5’-GAAGTATACGGTGAGGCGGG-3’

*GABA-B-R1* (*Drosophila*) 173 bp

Forward; 5’-CTCAGGGCGATCGTATTGCT-3’

Reverse; 5’-GGTGCGTAGAACATGGGTGA-3’

*GABA-B-R2* (*Drosophila*) 178 bp

Forward; 5’-TGGCGGGTGCATTCGATATT-3’

Reverse; 5’-TCTCGTGATGCAAGGGTTCC-3’

*GABA-B-R3* (*Drosophila*) 181 bp

Forward; 5’-AATTCGCACAGCAATCTGCC-3’

Reverse; 5’-ACAGCTCAAAGAGTCCGAGC-3’

*sima* (*Drosophila*) 213 bp

Forward; 5’-AGCCCAATCTGCCGCCAACC-3’

Reverse; 5’-TGGAGGCCAGGTGGTGGGAC-3’

*Srl* (*Drosophila*) 136 bp

Forward; 5’-GGATTCACGAATGCTAAATGTGTTCC-3’

Reverse; 5’-GATGGGTAGGATGCCGCTCAG-3’

*Nup133* (*Drosophila*) 180 bp

Forward; 5’-GGGCCATGACGTACTGGAAA-3’

Reverse; 5’-ATCTTGTGCCGCATGTCGTA-3’

*Marf* (*Drosophila*) 199 bp

Forward; 5’-CGCTATCCCGGTTCAACTCA-3’

Reverse; 5’-TGCAGGGCGTTGATAAAGGT-3’

*Opa1* (*Drosophila*) 196 bp

Forward; 5’-GCTACGATACGGGCTACACC-3’

Reverse; 5’-CGGAACTGAGCCACATGGTA-3’

*Fis1* (*Drosophila*) 184 bp

Forward; 5’-CATCCAGATGGAAGGCGTGA-3’

Reverse; 5’-CCACCATACCCTTTGCCACT-3’

*Drp1* (*Drosophila*) 166 bp

Forward; 5’-TACTGCTGCACAATCGAGGG-3’

Reverse; 5’-CATTCCGGATGGCTGTCAGT-3’

*tau* (Human) 184 bp

Forward; 5’-ATGCACCAAGACCAAGAGGG-3’

Reverse; 5’-TCGTTTTACCATCAGCCCCC-3’

*GAPDH1* (*Drosophila*) 109 bp

Forward; 5’-GACGAAATCAAGGCTAAGGTCG-3’

Reverse; 5’-AATGGGTGTCGCTGAAGAAGTC-3’

### Construction of *Drosophila wfs1* expression vector

cDNA encoding the full length of *Drosophila wfs1* (isoform C) were cloned by PCR from *Drosophila* genome from UAS-*wfs1* fly, and HA tag was fused at the C-terminus. The cDNA was subcloned into a pUAST vector.

### Cell culture and immunostaining

*Drosophila* S2 cells were maintained in Schneider's *Drosophila* media (Thermo Fisher Scientific) supplemented with 10% FBS in a 25°C incubator. S2 cells were transfected with actin-GAL4 and UAS-*wfs1*-HA plasmids using HilyMax (Dojindo) following the manufacturer’s protocol. Cells were fixed in 4% paraformaldehyde in phosphate-buffered saline (PBS) at 48 h after transfection and stained with anti-HA (Santa Cruz Biotechnology) or anti-KDEL (Enzo Life Sciences) antibody. Antibodies were detected by anti-rabbit IgG conjugated Alexa 488 (Abcam) or ant-mouse IgG conjugated Alexa 647 (Abcam). ER and mitochondria were labeled by Concanavalin A (ConA) conjugated Alexa 647 (Thermo Fisher Scientific) and Mitotracker (Thermo Fisher Scientific) respectively. The stained cells were analyzed using a confocal microscope (Carl Zeiss, LSM 780).

### ATP assay

ATP contents in fly brains were analyzed using ATP Bioluminescence Assay Kit CLSII (Roche) according to the manufacturer’s protocol. Briefly, dissected fly brains were homogenized in Tris-EDTA buffer and boil at 100°C for 2 min to inactivate ATPase. Samples were centrifuged at 16,000 × g for 5 min and the supernatant was used for ATP assay. ATP levels were calculated by standard curves and normalized by protein levels.

### Confocal analysis for mitochondrial distribution

Mitochondrial distribution in fly neuron was analyzed as described previously [65]. Fly brains were dissected in cold PBS, fixed in PBS containing 4% paraformaldehyde, and then placed under vacuum in PBS containing 4% paraformaldehyde and 0.25% Triton X-100. The fluorescence intensity in the mushroom body regions was analyzed using a confocal microscope (Carl Zeiss, LSM 780) and quantified using Image J (NIH).

### Statistics

All results were expressed as mean ± s.d. (for climbing assay) or mean ± s.e.m. Unpaired Student’s *t*-test (Excel, Microsoft) was used to determine statistical significance as indicated in the figure legends. Mann-Whitney *U*-test was used to determine statistical significance for righting reflex assay. Kaplan-Meier survival analyses with log-rank tests (SigmaPlot 11.0, Systat Software Inc.) were used to determine statistical significance for lifespan analysis. One-way ANOVA followed by Tukey's post hoc test was used to determine statistical significance as indicated in the figure legends. * indicates *p* < 0.05, ** indicates *p* < 0.01 and *** indicates *p* < 0.001 throughout the manuscript.

## Supporting information

S1 Table Genotype and age of flies used in this study. (DOCX)

S1 Fig Knockdown of *wfs1*, a fly homolog of *WFS1*, in the nervous system causes behavioral deficits and shortens lifespan of flies. (**A**) mRNA expression levels of *wfs1* in heads of flies carrying the RNAi transgene (GD line; *wfs1* RNAi^GD^) targeting *wfs1* were analyzed by qRT-PCR. n = 4, ***p < 0.001 by Student’s *t*-test. (**B**) Knockdown of *wfs1* in neurons induced age-dependent locomotor deficits as revealed by climbing assay. Average percentages of flies that climbed to the top (white), climbed to the middle (light gray), or stayed at the bottom (dark gray) of the vials. Percentages of flies that stayed at the bottom were subjected to statistical analyses. The experiments were repeated two times and representative data is shown. n = 5, *p < 0.05 and ***p < 0.001 by Student’s *t*-test. (**C**) Knockdown of *wfs1* in both neurons and glial cells (*elav-Repo*) significantly shortened lifespan of flies (n = 132, *wfs1* RNAi group or 233, *Luciferase* RNAi group). The lifespans of flies were determined by Kaplan-Meier survival analysis with log-rank test and statistical significance was indicated in the figure. Genotypes and ages of flies are described in S1 Table.
(TIF)

S2 Fig Mutations in *wfs1* gene cause behavioral deficits and shorten lifespan of flies. (**A**-**B**) The mRNA expression levels of *wfs1* and *Nup133* in *wfs1* mutant PiggyBac lines, *wfs1*^e03461/e03461^ (**A**) and *wfs1*^LL07290/LL07290^ (**B**) were analyzed by qRT-PCR. n = 4, **p < 0.01 and ***p < 0.001 by Student’s *t*-test. (**C**) The flies carrying a heterozygous mutation (*wfs1*^LL07290/+^) and homozygous mutation (*wfs1*^LL07290/LL07290^) of *wfs1* induced age-dependent locomotor deficits as revealed by climbing assay. Average percentages of flies that climbed to the top (white), climbed to the middle (light gray), or stayed at the bottom (dark gray) of the vials. Percentages of flies that stayed at the bottom were subjected to statistical analyses. n = 5 independent experiments, *p < 0.05, **p < 0.01 and ***p < 0.001 by Student’s *t*-test. (**D**) A heterozygous mutation (*wfs1*^LL07290/+^) and homozygous mutation (*wfs1*^LL07290/LL07290^) of *wfs1* shortened the lifespan of flies. The lifespans of flies were determined by Kaplan-Meier survival analysis with log-rank test, and Holm-Sidak method was used for multiple comparison analysis (n = 316, Control, n = 314, *wfs1*^LL07290/+^, n = 92, *wfs1*^LL07290/LL07290^). The statistical significance was indicated in the figure. Genotypes and ages of flies are described in S1 Table.
(TIF)

S3 Fig Predicted amino acid sequence of wfs1 in the flies carrying homozygous MiMIC insertions in the *wfs1* gene. Alignment of amino acid sequences predicted from the DNA sequence of *wfs1* with the MiMIC insertion (wfs1_MI140411) and that of wild-type *wfs1* from NCBI database (wfs1_NP_001189267.1).
(TIF)

S4 Fig *Drosophila* wfs1 co-localizes with ER and mitochondria markers in *Drosophila* S2 cells. (**A-C**) Localization of wfs1-HA in *Drosophila* Schneider 2 (S2) cells. S2 cells were transiently transfected with wfs1-HA and double stained with anti-HA tag antibody (for wfs1-HA) and anti-KDEL (ER marker) antibody (**A**), Concanavalin A (ConA) conjugated Alexa Fluor (ER marker) (**B**) or Mitotracker (mitochondrial marker) (**C**). Nuclei were counterstained with DAPI. Samples were analyzed by confocal microscopy. Scale bars: 5 μm.
(TIF)

S5 Fig ER stress induces *wfs1* expression in *Drosophila*. (**A**) Neuronal expression of misfolding prone amyloid-β42 peptides (Aβ42) in the ER increases mRNA expression levels of *Xbp1-RB*, *PEK* and *Hsc70-3* in fly heads, as determined by qRT-PCR. n = 4, **p < 0.01 and ***p < 0.001 by Student’s *t*-test. (**B**) mRNA levels of *wfs1* were increased in fly heads expressing Aβ42, as determined by qRT-PCR. n = 4, **p < 0.01 by Student’s *t*-test. Genotypes and ages of flies are described in S1 Table.
(TIF)

S6 Fig Neuronal knockdown of *wfs1* does not induce mitochondrial dysfunctions. **(A)** Double knockdown of *wfs1* in both neurons and glial cells did not affect the level of total ATP content. Adult fly brains expressing RNAi transgene for *wfs1* in neurons and glial cells were subjected to the experiment. n = 3 independent experiments, no significant difference. (**B**) mRNA levels of genes related to mitochondrial fission and fusion were not altered in fly brains with neuronal and glial knockdown of *wfs1*, as determined by qRT-PCR. n = 4, *p < 0.05 by Student’s *t*-test. (**C**) Fly heads expressing RNAi transgene for *wfs1* were subjected to western blotting with anti-VDAC1, anti-NDUFS3 and anti-MnSOD antibodies. Tubulin was used as the loading control. n = 4, no significant difference. (**D**) A schematic diagram of mitochondria-targeted GFP (mito-GFP) and mito-GFP signals in the mushroom body structure in the fly brain. A GFP is fused to a mitochondria-targeting signal of human cytochrome *c* oxidase subunit VIII (cCoxVIII) (left panel). Fly brains expressing mito-GFP in neurons were dissected and confocal images were captured. Lobe tips (Axons), Kenyon cells (Cell bodies) and Calyx (Dendrites) are indicated (right panel). (**E**) Neuronal knockdown of *wfs1* did not disrupt mitochondrial distribution in fly neurons. Representative images show the mito-GFP distribution in the mushroom structure in the fly brain at 7-, 21-, and 42-day-old. Signal intensities of mito-GFP in the axons, dendrites and cell bodies of the mushroom body structure in control or *wfs1* RNAi fly brains were quantified. n = 8–10 hemispheres, ***p < 0.001 by Student’s *t*-test. (**F**) A heterozygous *Opa1* mutation (*Opa1*^*s3475*^) did not exacerbated age-dependent locomotor deficits caused by neuronal knockdown of *wfs1*. Average percentages of flies that climbed to the top (white), climbed to the middle (light gray), or stayed at the bottom (dark gray) of the vials. Percentages of flies that stayed at the bottom were subjected to statistical analyses. n = 3–5 independent experiments, no significant difference. Genotypes and ages of flies are described in S1 Table.
(TIF)

S7 Fig Neuronal knockdown of *wfs1* does not induce lysosome/autophagy defects in the fly brains. Fly heads expressing RNAi transgene for *wfs1* or *mcherry* in neurons were subjected to western blotting with anti-Atg8 and anti-ref(2)P antibodies. Tubulin was used as a loading control. The ratios of Atg8-II/Atg8-I were analyzed. Atg8; n = 12, ref(2)P; n = 4, no significant difference. Genotypes and ages of flies are described in S1 Table.
(TIF)

S8 Fig Neither glutamate release inhibitor riluzole nor anticholinergic agent orphenadrine alter the age-dependent locomotor deficits caused by neuronal knockdown of *wfs1*. (**A**-**B**) Glutamate release inhibitor riluzole (**A**) and anticholinergic agent orphenadrine (**B**) did not alter age-dependent locomotor deficits in both *wfs1* knockdown background (left panels) and control background (right panels). Average percentages of flies that climbed to the top (white), climbed to the middle (light gray), or stayed at the bottom (dark gray) of the vials. Percentages of flies that stayed at the bottom were subjected to statistical analyses. n = 3 independent experiments, no significant difference. (**C**) Anticholinergic agent orphenadrine did not alter the lifespan of flies with neuronal and glial knockdown of *wfs1*. The lifespans of flies were determined by Kaplan-Meier survival analysis with log-rank test and Holm-Sidak method was used for multiple comparison analysis (n = 109, *mcherry* RNAi with Orphenadrine 0 mM, n = 107, *mcherry* RNAi with Orphenadrine 1 mM, n = 103, *wfs1* RNAi with Orphenadrine 0 mM, n = 102, *wfs1* RNAi with Orphenadrine 1 mM). The statistical significance was indicated in the figure. Genotypes and ages of flies are described in S1 Table.
(TIF)

S9 Fig Overexpression of *wfs1* does not suppress the axon degeneration caused by human tau. (**A**) mRNA levels of *tau* were determined by qRT-PCR. n = 4, no significant difference by one-way ANOVA with Tukey’s post hoc test. (**B**) Overexpression of *wfs1* did not suppressed retinal degeneration induced by ectopic overexpression of human tau. Scale bar, 200 μm. (**C**) Overexpression of *wfs1* did not suppress axon degeneration in the lamina caused by ectopic overexpression of human tau. Representative images show the lamina in paraffin-embedded head section with hematoxylin and eosin (HE) staining from 7-day-old flies. Scale bars: 200 μm. Percentages of vacuole areas in the lamina (indicated by arrowheads in the images) are shown. n = 12 hemispheres, no significant difference by one-way ANOVA with Tukey’s post hoc test. Genotypes and ages of flies are described in S1 Table.
(TIF)

S10 Fig Neuronal and glial knockdown of *wfs1* does not alter the levels of neurotrophic factor, MANF, in fly brains. Fly heads expressing RNAi transgene for *wfs1* or *mcherry* in both neurons and glial cells were subjected to western blotting with anti-MANF antibody. Tubulin was used as the loading control. n = 4, no significant difference. Genotypes and ages of flies are described in S1 Table.
(TIF)
