# Supplementary material for: Knockdown of wfs1, a fly homolog of Wolfram syndrome 1, in the nervous system increases susceptibility to age- and stress-induced neuronal dysfunction and degeneration in Drosophila
Source: PLoS Genet. 2018 Jan 22;14(1):e1007196. doi: 10.1371/journal.pgen.1007196 (PMC5794194; doi:10.1371/journal.pgen.1007196)
Supplement: S1 Table — (DOCX) [file pgen.1007196.s001.docx]

**S1 Table. Genotype and age of flies used in this study.**

| **Figure #** | **Label of flies** | **Genotype** | **Age** |
| --- | --- | --- | --- |
| Fig 1 | 7 day-old and 30 day-old | elav-GAL4/Y | Fig 1**A** (described in figure). |
|  | Control | elav-GAL4/Y;;UAS-Luciferase/+ | Fig 1**C** (7d), **D**, **E**, **F** (described in figure). |
|  | *mcherry* RNAi | elav-GAL4/Y;;UAS-mcherry RNAi/+ | Fig 1**D** (described in figure). |
|  | *wfs1* RNAi | elav-GAL4/Y;UAS-wfs1 RNAi/+ | Fig.1**C** (7d), **D**, **E**, **F** (described in figure). |
| Fig 2 | Control (elav-Repo) | elav-GAL4/Y;;UAS-Luciferase/Repo-GAL4 | Fig 2**A**, **B** (described in figure). |
|  |  | elav-GAL4/Y;;Repo-GAL4/+ | Fig 2**C** (described in figure). |
|  | *wfs1* RNAi (elav-Repo) | elav-GAL4/Y;UAS-wfs1 RNAi/+;Repo-GAL4/+ | Fig 2**A**, **B**, **C** (described in figure). |
|  | Control (Repo) | +/Y;;UAS-Luciferase/Repo-GAL4 | Fig 2**D**, **E**, **F** (described in figure). |
|  | *wfs1* RNAi (Repo) | +/Y;UAS-wfs1 RNAi/+;Repo-GAL4/+ | Fig 2**D**, **E**, **F** (described in figure). |
| Fig 3 | Control | +/Y | Fig 3**A** (33d), **C**, **D** (described in figure). |
|  | *wfs1*^MI14041/MI14041^ | +/Y;;wfs1^MI14041^/wfs1^MI14041^ | Fig 3**A** (33d), **C**, **D** (described in figure). |
| Fig 4 | Control (elav) | elav-GAL4/Y;;UAS-Luciferase/+ | Fig 4**A** (30d). |
|  | *wfs1* RNAi (elav) | elav-GAL4/Y;UAS-wfs1 RNAi/+ | Fig 4**A** (30d). |
|  | Control (elav-Repo) | elav-GAL4/Y;;UAS-Luciferase/Repo-GAL4 | Fig 4**B** (14d). |
|  | *wfs1* RNAi (elav-Repo) | elav-GAL4/Y;UAS-wfs1 RNAi/+;Repo-GAL4/+ | Fig 4**B** (14d). |
|  | Control | +/Y | Fig 4**C** (33d). |
|  | *wfs1*^MI14041/MI14041^ | +/Y;;wfs1^MI14041^/wfs1^MI14041^ | Fig 4**C** (33d). |
| Fig 5 | Control and H_2_O_2_ exposure | elav-GAL4/Y | Fig 5**A** (8d). |
|  | *mcherry* RNAi | elav-GAL4/Y;;UAS-mcherry RNAi/+ | Fig 5**B** (11-15d), **C** (described in figure). |
|  | *wfs1* RNAi | elav-GAL4/Y;UAS-wfs1 RNAi/+ | Fig 5**B** (11-15d), **C** (described in figure). |
| Fig 6 | *mcherry* RNAi (elav) | elav-GAL4/Y;;UAS-mcherry RNAi/+ | Fig 6**A** (described in figure). |
|  | *wfs1* RNAi (elav) | elav-GAL4/Y;UAS-wfs1 RNAi/+ | Fig 6**A** (described in figure), **D** (21d). |
|  | *mcherry* RNAi (elav-Repo) | elav-GAL4/Y;;UAS-mcherry RNAi/Repo-GAL4 | Fig 6**A** (described in figure), **C** (14d), **H** (described in figure). |
|  | *wfs1* RNAi (elav-Repo) | elav-GAL4/Y;UAS-wfs1 RNAi/+;Repo-GAL4/+ | Fig 6**A** (described in figure), **C** (14d), **H** (described in figure). |
|  | Control | +/Y | Fig 6**B** (described in figure). |
|  | *wfs1*^LL07290/LL07290^ | +/Y;;wfs1^LL07290^/wfs1^LL07290^ | Fig 6**B**, **I** (described in figure). |
|  | Control (elav) | elav-GAL4/Y;;UAS-Luciferase/+ | Fig 6**D** (21d) |
|  | *Eaat1 +/+* (Control background) | elav-GAL4/Y;Eaat1^PE^/+ | Fig 6**E** (described in figure), **F**, **G** (30d). |
|  | *Eaat1 +/-* (Control background) | elav-GAL4/Y;Eaat1^SM2^/+ | Fig 6**E** (described in figure), **F**, **G** (30d). |
|  | *Eaat1 +/+* (*wfs1* knockdown background) | elav-GAL4/Y;Eaat1^PE^/UAS-wfs1 RNAi | Fig 6**E** (described in figure), **F**, **FG** (30d). |
|  | *Eaat1 +/-* (*wfs1* knockdown background) | elav-GAL4/Y;Eaat1^SM2^/UAS-wfs1 RNAi | Fig 6**E** (described in figure), **F**, **G** (30d). |
| Fig 7 | Control | +/+;GMR-GAL4/+;UAS-mcherry RNAi/+ | Fig 7**A**, **B**, **E** (7d). |
|  | tau | +/+;GMR-GAL4, UAS-tau/+;UAS-mcherry RNAi/+ | Fig 7**A**, **B**, **E** (7d). |
|  |  | +/+;GMR-GAL4, UAS-tau/+ | Fig 7**C** (described in figure), **D** (10d). |
|  | tau/*wfs1* RNAi | +/+;GMR-GAL4, UAS-tau/UAS-wfs1 RNAi | Fig 7**C** (described in figure), **D** (10d), **E** (7d). |
|  | Control | +/+;GMR-GAL4/+ | Fig 7**C** (described in figure). |
|  | *wfs1* RNAi | +/+;GMR-GAL4/UAS-wfs1 RNAi | Fig 7**C** (described in figure). |
| S1 Fig | Control | elav-GAL4/Y;UAS-Luciferase RNAi/+ | S1 **A** (10d), **B** (described in figure). |
|  | *wfs1* RNAi^GD^ | elav-GAL4/Y;;UAS-wfs1 RNAi/+ | S1 **A** (10d), **B** (described in figure). |
|  | *Luciferase* RNAi (elav-Repo) | elav-GAL4/Y;UAS-Luciferase RNAi/+;Repo-GAL4/+ | S1 **C** (described in figure). |
|  | *wfs1* RNAi^GD^ (elav-Repo) | elav-GAL4/Y;;UAS-wfs1 RNAi/Repo-GAL4 | S1 **C** (described in figure). |
| S2 Fig | Control | +/Y | S2 **A** (7-10d), **B** (3d), **C**, **D** (described in figure). |
|  | *wfs1*^e03461/e03461^ | +/Y ;;wfs1^e03461^/wfs1^e03461^ | S2 **A** (7-10d). |
|  | *wfs1*^LL07290/+^ | +/Y ;;wfs1^LL07290^/+ | S2 **B** (3d), **C**, **D** (described in figure). |
|  | *wfs1*^LL07290/LL07290^ | +/Y ;;wfs1^LL07290^/wfs1^LL07290^ | S2 **B** (3d), **C**, **D** (described in figure). |
| S5 Fig | Control | elav-GAL4/Y | S5 **A**, **B** (7d). |
|  | Aβ42 | elav-GAL4/Y;UAS-Aβ42/+ | S5 **A**, **B** (7d). |
| S6 Fig | Control (elav-Repo) | elav-GAL4/Y;;UAS-Luciferase/Repo-GAL4 | S6 **A** (7d), **B** (14d). |
|  | *wfs1* RNAi (elav-Repo) | elav-GAL4/Y;UAS-wfs1 RNAi/+;Repo-GAL4/+ | S6 **A** (7d), **B** (14d). |
|  | Control | elav-GAL4/Y;;UAS-Luciferase/+ | S6 **C** (30d). |
|  |  | elav-GAL4/Y;UAS-mitoGFP/+ | S6 **E** (described in figure). |
|  | *wfs1* RNAi (elav) | elav-GAL4/Y;UAS-wfs1 RNAi/+ | S6 **C** (30d). |
|  |  | elav-GAL4/Y;UAS-mitoGFP/UAS-wfs1 RNAi | S6 **E** (described in figure). |
|  | Control | elav-GAL4/Y | S6 **F** (described in figure). |
|  | *Opa1*^s3475^ | elav-GAL4/Y;Opa1^s3475^/+ | S6 **F** (described in figure). |
|  | Control (*wfs1* knockdown background) | elav-GAL4/Y;UAS-wfs1 RNAi/+ | S6 **F** (described in figure). |
|  | *Opa1*^s3475^ (*wfs1* knockdown background) | elav-GAL4/Y;UAS-wfs1 RNAi/Opa1^s3475^ | S6 **F** (described in figure). |
| S7 Fig | Control (elav) | elav-GAL4/Y | S7 (30d). |
|  | *mcherry* RNAi (elav) | elav-GAL4/Y;;UAS-mcherry RNAi/+ | S7 (30d). |
|  | *wfs1* RNAi (elav) | elav-GAL4/Y;UAS-wfs1 RNAi/+ | S7 (30d). |
| S8 Fig | Control, Riluzole 0.2 mM, 1 mM (*wfs1* knockdown background) (elav) | elav-GAL4/Y;UAS-wfs1 RNAi/+ | S8 **A** (described in figure). |
|  | Control, Riluzole 0.2 mM, 1 mM (Control background) (elav) | elav-GAL4/Y;;UAS-mcherry RNAi/+ | S8 **A** (described in figure). |
|  | Control, Orphenadrine 10 μM, 100 μM (*wfs1* knockdown background) (elav) | elav-GAL4/Y;UAS-wfs1 RNAi/+ | S8 **B** (described in figure). |
|  | Control, Orphenadrine 10 μM, 100 μM (Control background) (elav) | elav-GAL4/Y;;UAS-mcherry RNAi/+ | S8 **B** (described in figure). |
|  | *mcherry* RNAi (elav-Repo) | elav-GAL4/Y;;UAS-mcherry RNAi/Repo-GAL4 | S8 **C** (described in figure). |
|  | *wfs1* RNAi (elav-Repo) | elav-GAL4/Y;UAS-wfs1 RNAi/+;Repo-GAL4/+ | S8 **C** (described in figure). |
| S9 Fig | tau (GMR) | +/+;GMR-GAL4, UAS-tau/+ | S9 **A**, **B** (8d), **C** (described in figure). |
|  | tau/*wfs1 (II)* (GMR) | +/+;GMR-GAL4, UAS-tau/UAS-wfs1 | S9 **A**, **B** (8d), **C** (described in figure). |
|  | tau/*wfs1 (III)* (GMR) | +/+;GMR-GAL4, UAS-tau/+;UAS-wfs1/+ | S9 **A**, **B** (8d), **C** (described in figure). |
| S10 Fig | Control | elav-GAL4/Y;;Repo-GAL4/+ | S10 (16d). |
|  | *mcherry* RNAi (elav-Repo) | elav-GAL4/Y;;UAS-mcherry RNAi/Repo-GAL4 | S10 (16d). |
|  | *wfs1* RNAi (elav-Repo) | elav-GAL4/Y;UAS-wfs1 RNAi/+;Repo-GAL4/+ | S10 (16d). |
