# Supplementary figures and images for: Knockdown of wfs1, a fly homolog of Wolfram syndrome 1, in the nervous system increases susceptibility to age- and stress-induced neuronal dysfunction and degeneration in Drosophila
Source: PLoS Genet. 2018 Jan 22;14(1):e1007196. doi: 10.1371/journal.pgen.1007196 (PMC5794194; doi:10.1371/journal.pgen.1007196)

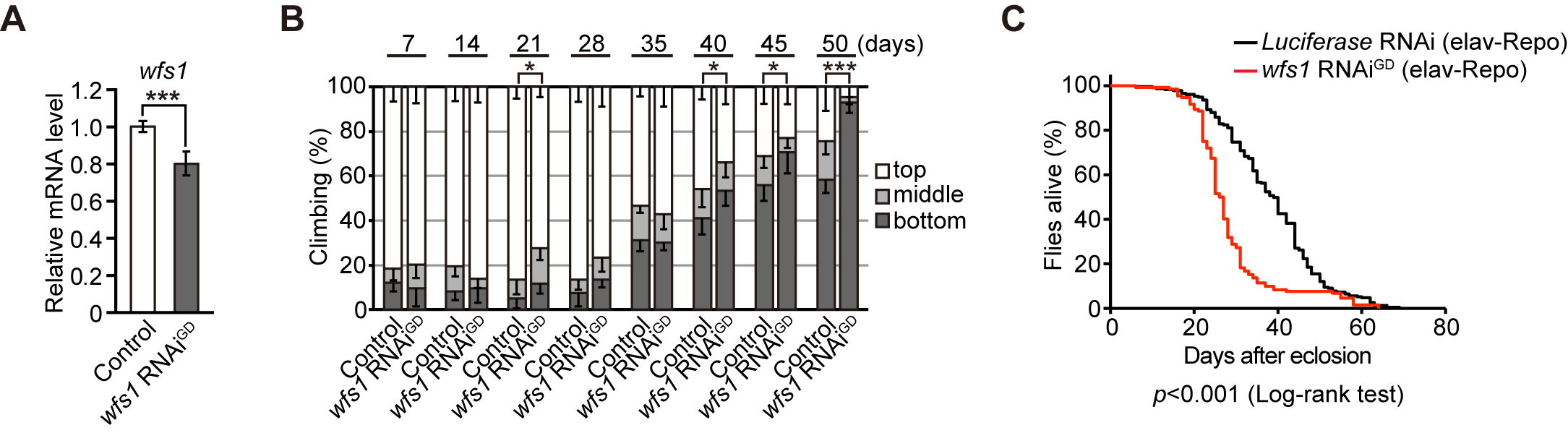

Supplement: S1 Fig — (A) mRNA expression levels of wfs1 in heads of flies carrying the RNAi transgene (GD line; wfs1 RNAiGD) targeting wfs1 were analyzed by qRT-PCR. n = 4, ***p < 0.001 by Student’s t-test. (B) Knockdown of wfs1 in neurons induced age-dependent locomotor deficits as revealed by climbing assay. Average percentages of flies that climbed to the top (white), climbed to the middle (light gray), or stayed at the bottom (dark gray) of the vials. Percentages of flies that stayed at the bottom were subjected to statistical analyses. The experiments were repeated two times and representative data is shown. n = 5, *p < 0.05 and ***p < 0.001 by Student’s t-test. (C) Knockdown of wfs1 in both neurons and glial cells (elav-Repo) significantly shortened lifespan of flies (n = 132, wfs1 RNAi group or 233, Luciferase RNAi group). The lifespans of flies were determined by Kaplan-Meier survival analysis with log-rank test and statistical significance was indicated in the figure. Genotypes and ages of flies are described in S1 Table. (TIF) [file pgen.1007196.s002.tif]

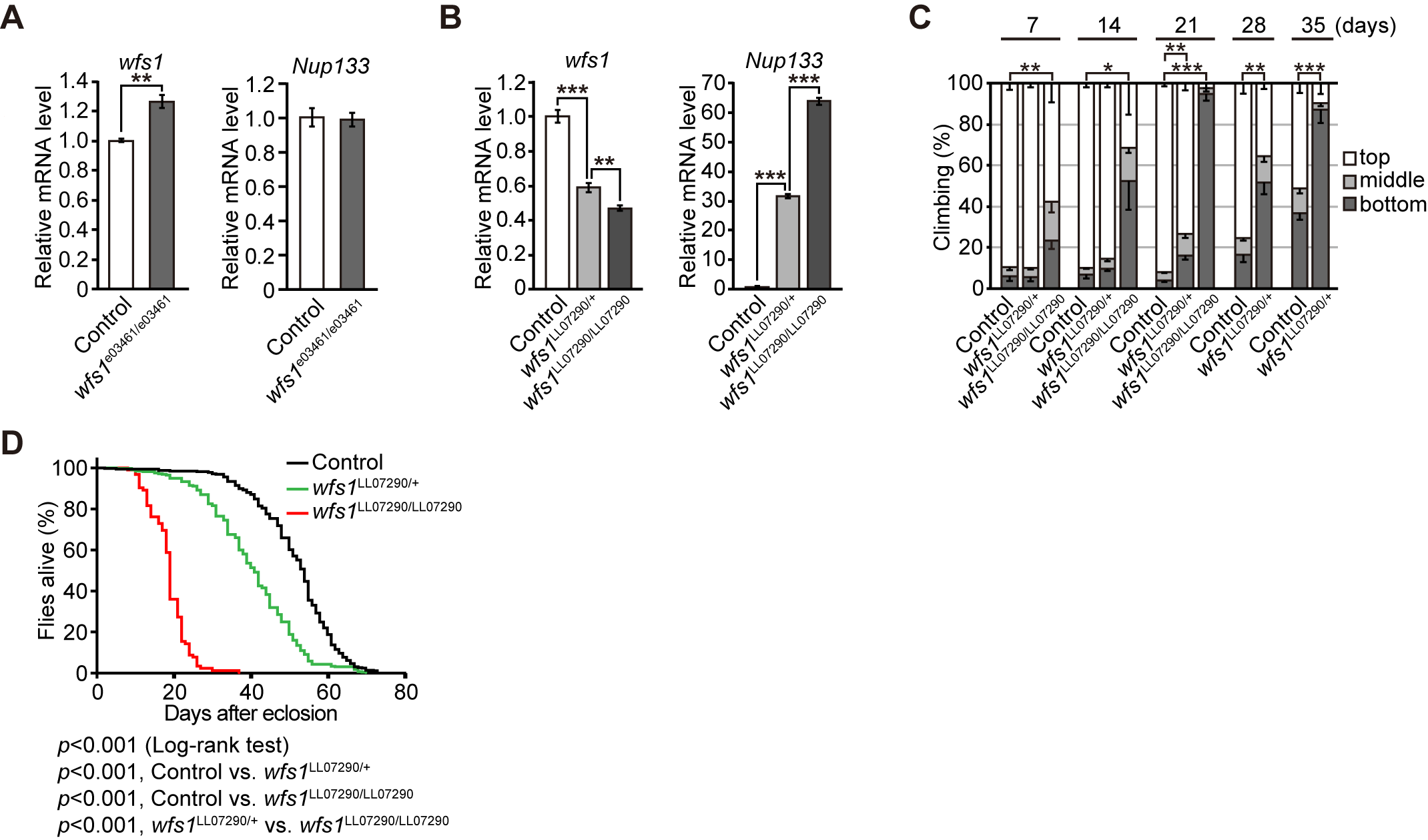

Supplement: S2 Fig — (A-B) The mRNA expression levels of wfs1 and Nup133 in wfs1 mutant PiggyBac lines, wfs1e03461/e03461 (A) and wfs1LL07290/LL07290 (B) were analyzed by qRT-PCR. n = 4, **p < 0.01 and ***p < 0.001 by Student’s t-test. (C) The flies carrying a heterozygous mutation (wfs1LL07290/+) and homozygous mutation (wfs1LL07290/LL07290) of wfs1 induced age-dependent locomotor deficits as revealed by climbing assay. Average percentages of flies that climbed to the top (white), climbed to the middle (light gray), or stayed at the bottom (dark gray) of the vials. Percentages of flies that stayed at the bottom were subjected to statistical analyses. n = 5 independent experiments, *p < 0.05, **p < 0.01 and ***p < 0.001 by Student’s t-test. (D) A heterozygous mutation (wfs1LL07290/+) and homozygous mutation (wfs1LL07290/LL07290) of wfs1 shortened the lifespan of flies. The lifespans of flies were determined by Kaplan-Meier survival analysis with log-rank test, and Holm-Sidak method was used for multiple comparison analysis (n = 316, Control, n = 314, wfs1LL07290/+, n = 92, wfs1LL07290/LL07290). The statistical significance was indicated in the figure. Genotypes and ages of flies are described in S1 Table. (TIF) [file pgen.1007196.s003.tif]

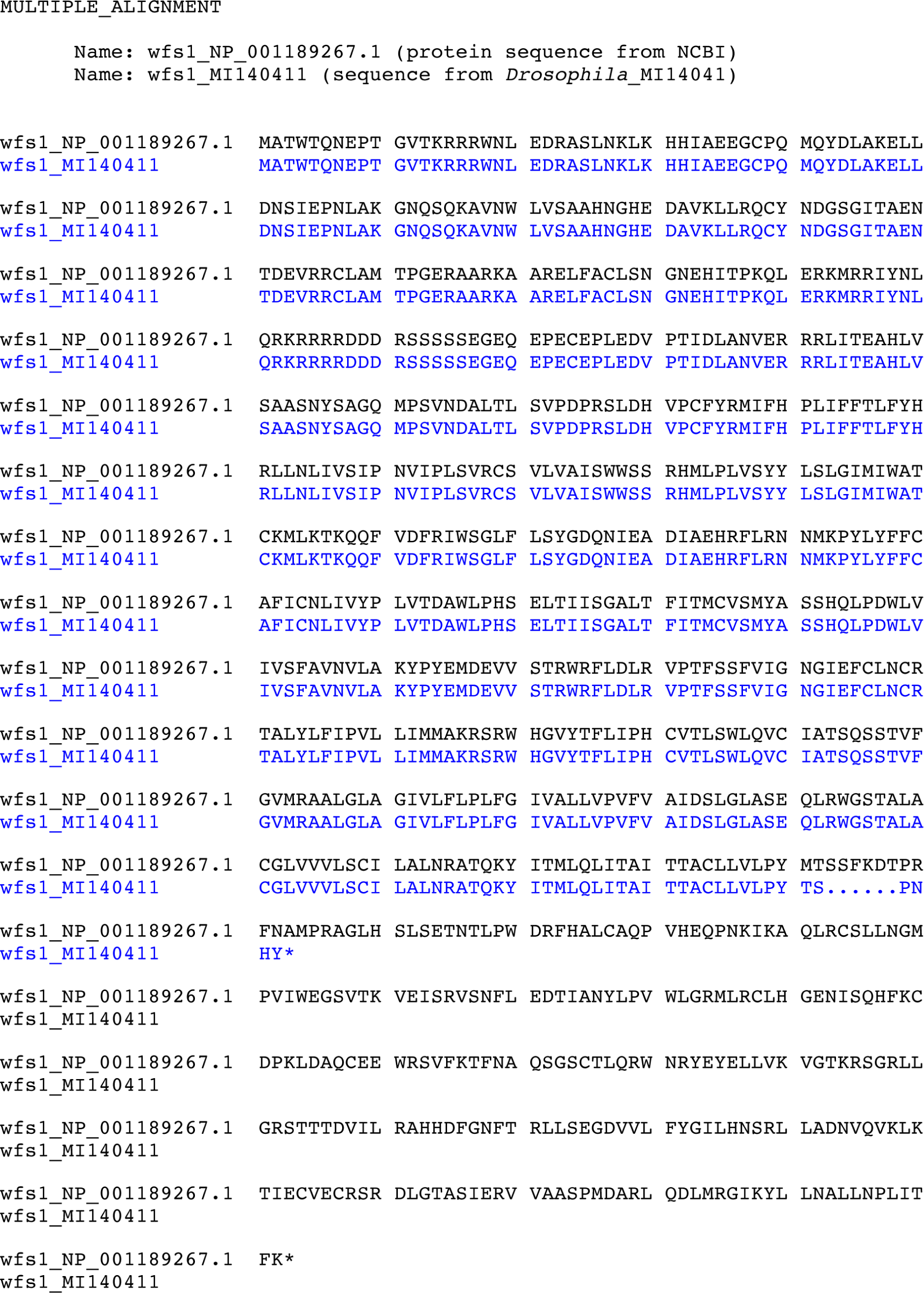

Supplement: S3 Fig — Alignment of amino acid sequences predicted from the DNA sequence of wfs1 with the MiMIC insertion (wfs1_MI140411) and that of wild-type wfs1 from NCBI database (wfs1_NP_001189267.1). (TIF) [file pgen.1007196.s004.tif]

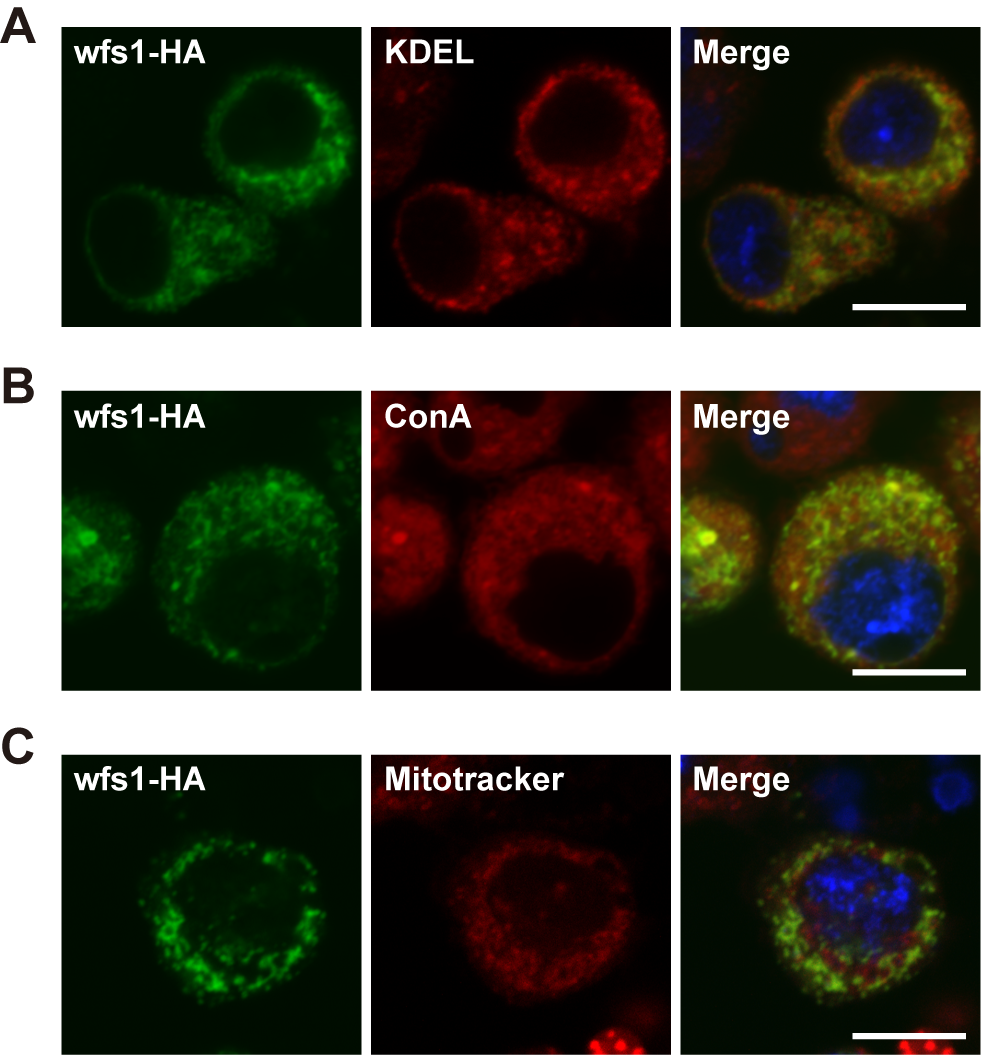

Supplement: S4 Fig — (A-C) Localization of wfs1-HA in Drosophila Schneider 2 (S2) cells. S2 cells were transiently transfected with wfs1-HA and double stained with anti-HA tag antibody (for wfs1-HA) and anti-KDEL (ER marker) antibody (A), Concanavalin A (ConA) conjugated Alexa Fluor (ER marker) (B) or Mitotracker (mitochondrial marker) (C). Nuclei were counterstained with DAPI. Samples were analyzed by confocal microscopy. Scale bars: 5 μm. (TIF) [file pgen.1007196.s005.tif]

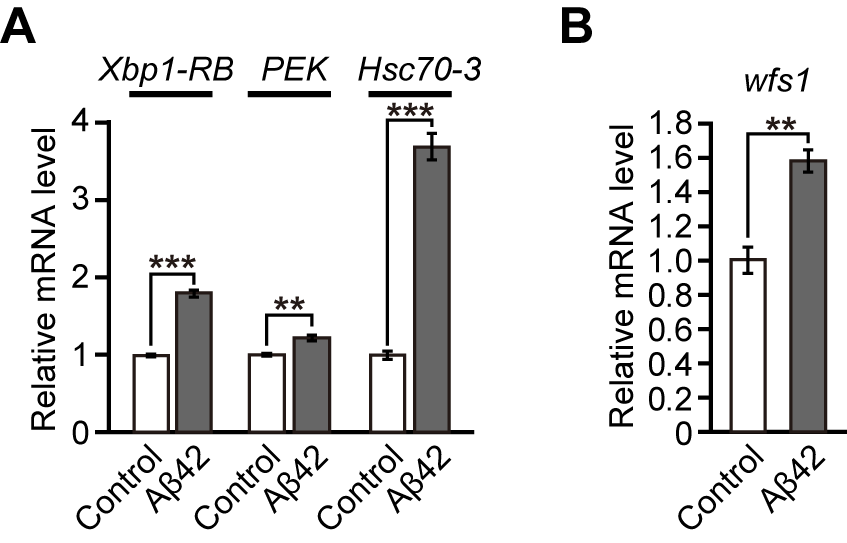

Supplement: S5 Fig — (A) Neuronal expression of misfolding prone amyloid-β42 peptides (Aβ42) in the ER increases mRNA expression levels of Xbp1-RB, PEK and Hsc70-3 in fly heads, as determined by qRT-PCR. n = 4, **p < 0.01 and ***p < 0.001 by Student’s t-test. (B) mRNA levels of wfs1 were increased in fly heads expressing Aβ42, as determined by qRT-PCR. n = 4, **p < 0.01 by Student’s t-test. Genotypes and ages of flies are described in S1 Table. (TIF) [file pgen.1007196.s006.tif]

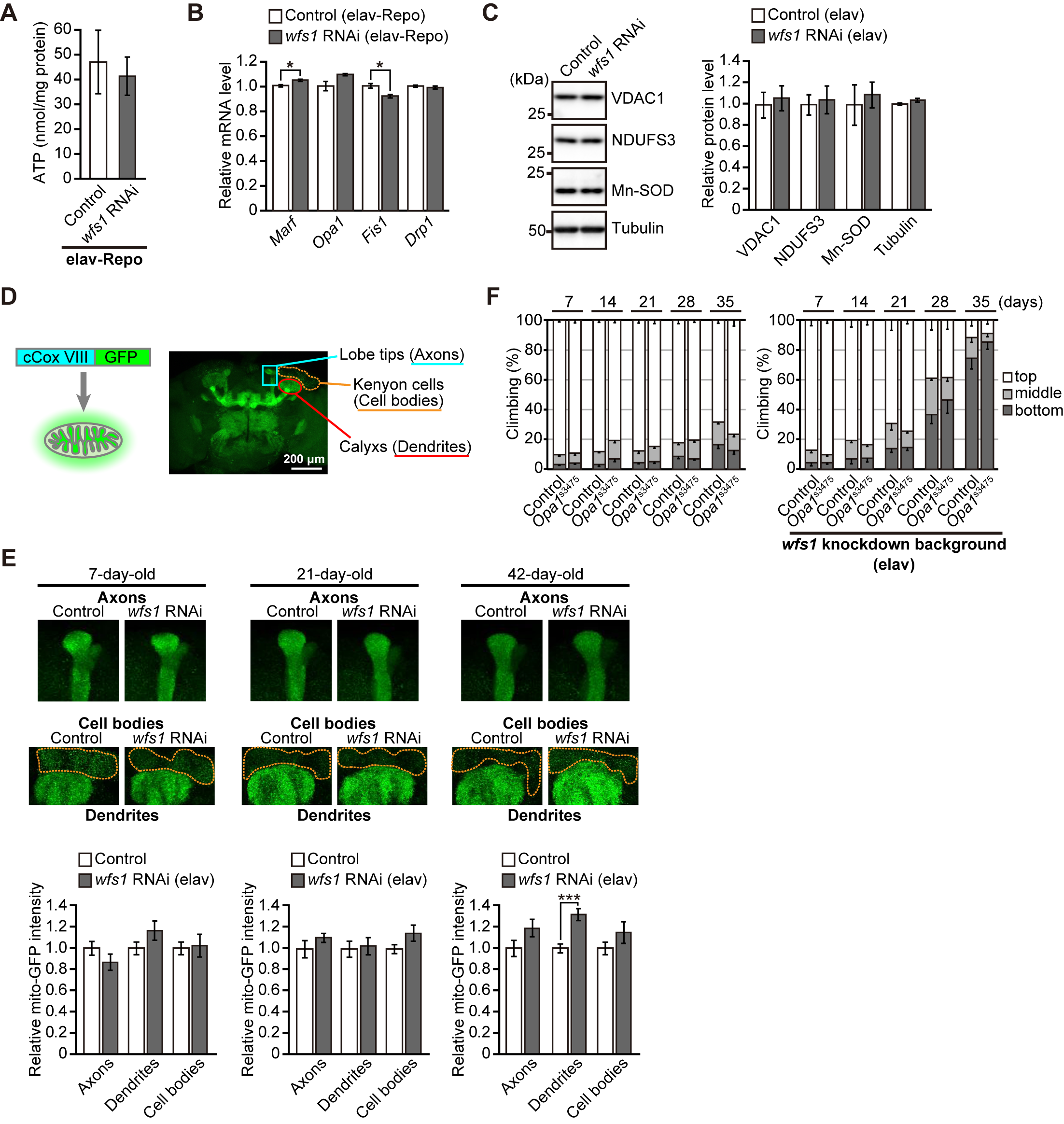

Supplement: S6 Fig — (A) Double knockdown of wfs1 in both neurons and glial cells did not affect the level of total ATP content. Adult fly brains expressing RNAi transgene for wfs1 in neurons and glial cells were subjected to the experiment. n = 3 independent experiments, no significant difference. (B) mRNA levels of genes related to mitochondrial fission and fusion were not altered in fly brains with neuronal and glial knockdown of wfs1, as determined by qRT-PCR. n = 4, *p < 0.05 by Student’s t-test. (C) Fly heads expressing RNAi transgene for wfs1 were subjected to western blotting with anti-VDAC1, anti-NDUFS3 and anti-MnSOD antibodies. Tubulin was used as the loading control. n = 4, no significant difference. (D) A schematic diagram of mitochondria-targeted GFP (mito-GFP) and mito-GFP signals in the mushroom body structure in the fly brain. A GFP is fused to a mitochondria-targeting signal of human cytochrome c oxidase subunit VIII (cCoxVIII) (left panel). Fly brains expressing mito-GFP in neurons were dissected and confocal images were captured. Lobe tips (Axons), Kenyon cells (Cell bodies) and Calyx (Dendrites) are indicated (right panel). (E) Neuronal knockdown of wfs1 did not disrupt mitochondrial distribution in fly neurons. Representative images show the mito-GFP distribution in the mushroom structure in the fly brain at 7-, 21-, and 42-day-old. Signal intensities of mito-GFP in the axons, dendrites and cell bodies of the mushroom body structure in control or wfs1 RNAi fly brains were quantified. n = 8–10 hemispheres, ***p < 0.001 by Student’s t-test. (F) A heterozygous Opa1 mutation (Opa1s3475) did not exacerbated age-dependent locomotor deficits caused by neuronal knockdown of wfs1. Average percentages of flies that climbed to the top (white), climbed to the middle (light gray), or stayed at the bottom (dark gray) of the vials. Percentages of flies that stayed at the bottom were subjected to statistical analyses. n = 3–5 independent experiments, no significant difference. Gen [file pgen.1007196.s007.tif]

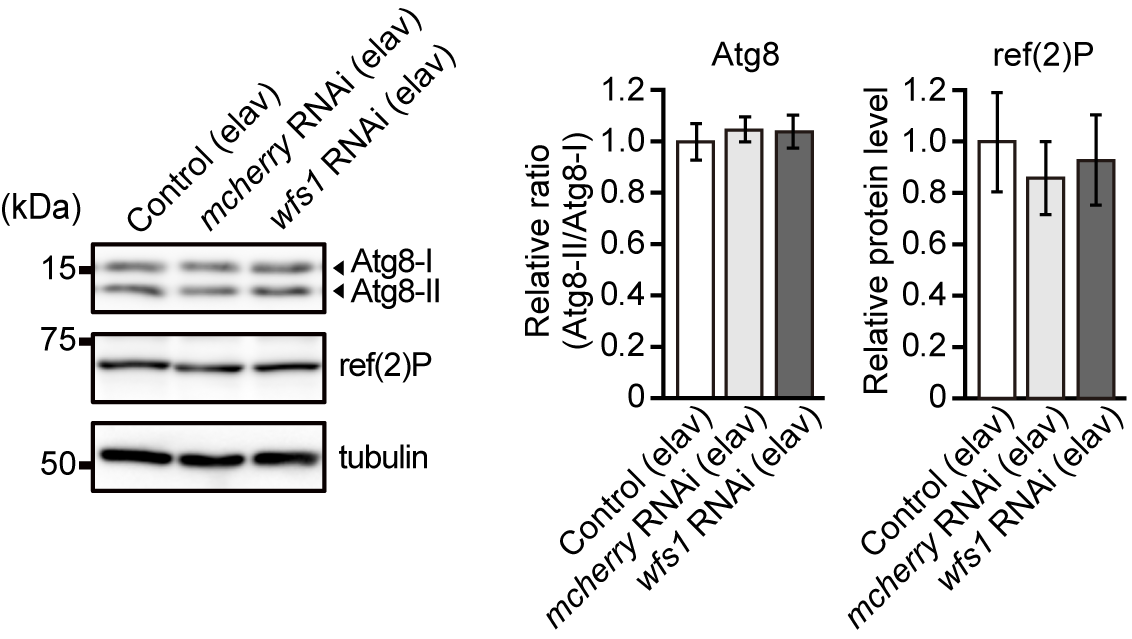

Supplement: S7 Fig — Fly heads expressing RNAi transgene for wfs1 or mcherry in neurons were subjected to western blotting with anti-Atg8 and anti-ref(2)P antibodies. Tubulin was used as a loading control. The ratios of Atg8-II/Atg8-I were analyzed. Atg8; n = 12, ref(2)P; n = 4, no significant difference. Genotypes and ages of flies are described in S1 Table. (TIF) [file pgen.1007196.s008.tif]

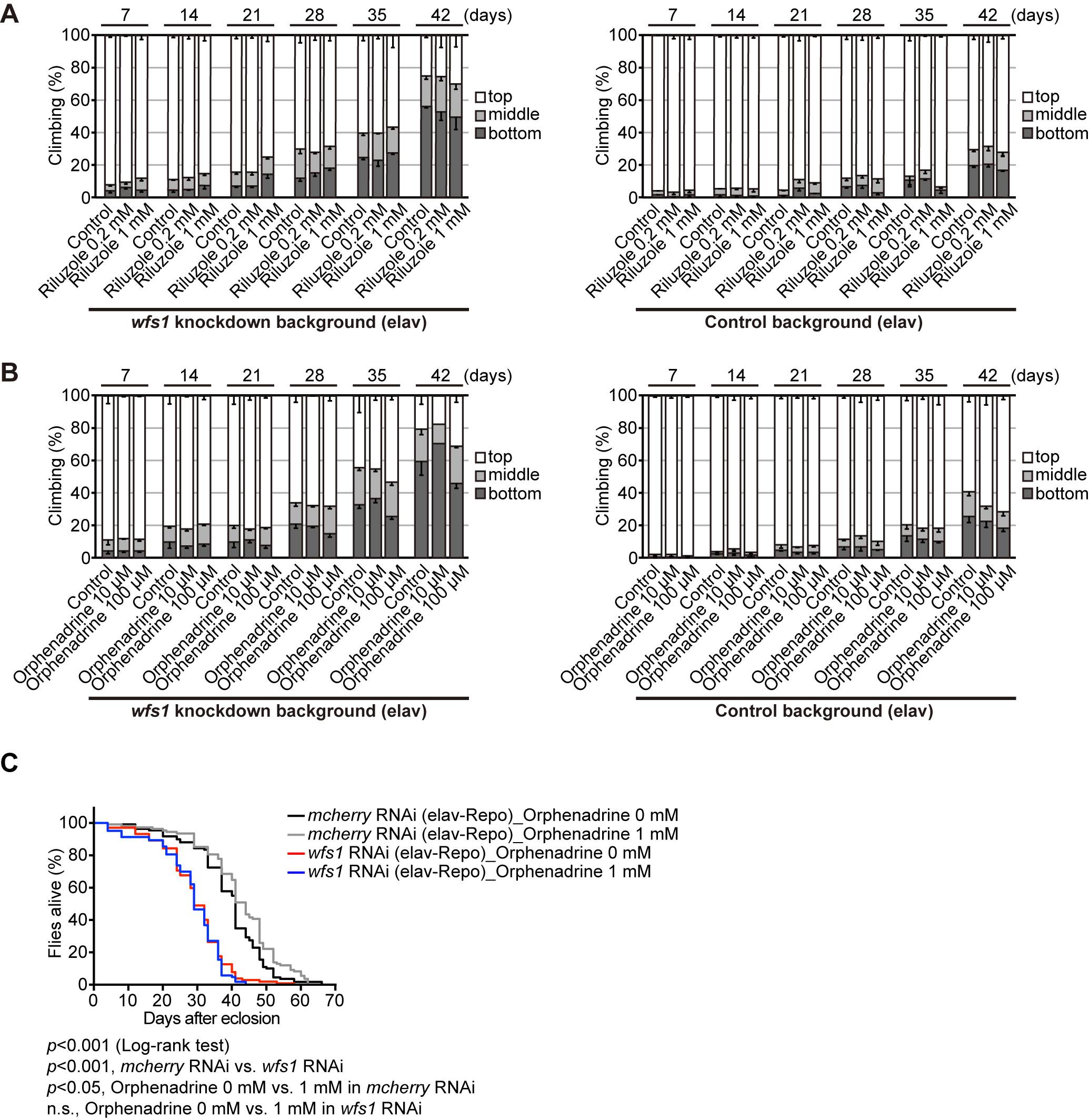

Supplement: S8 Fig — (A-B) Glutamate release inhibitor riluzole (A) and anticholinergic agent orphenadrine (B) did not alter age-dependent locomotor deficits in both wfs1 knockdown background (left panels) and control background (right panels). Average percentages of flies that climbed to the top (white), climbed to the middle (light gray), or stayed at the bottom (dark gray) of the vials. Percentages of flies that stayed at the bottom were subjected to statistical analyses. n = 3 independent experiments, no significant difference. (C) Anticholinergic agent orphenadrine did not alter the lifespan of flies with neuronal and glial knockdown of wfs1. The lifespans of flies were determined by Kaplan-Meier survival analysis with log-rank test and Holm-Sidak method was used for multiple comparison analysis (n = 109, mcherry RNAi with Orphenadrine 0 mM, n = 107, mcherry RNAi with Orphenadrine 1 mM, n = 103, wfs1 RNAi with Orphenadrine 0 mM, n = 102, wfs1 RNAi with Orphenadrine 1 mM). The statistical significance was indicated in the figure. Genotypes and ages of flies are described in S1 Table. (TIF) [file pgen.1007196.s009.tif]

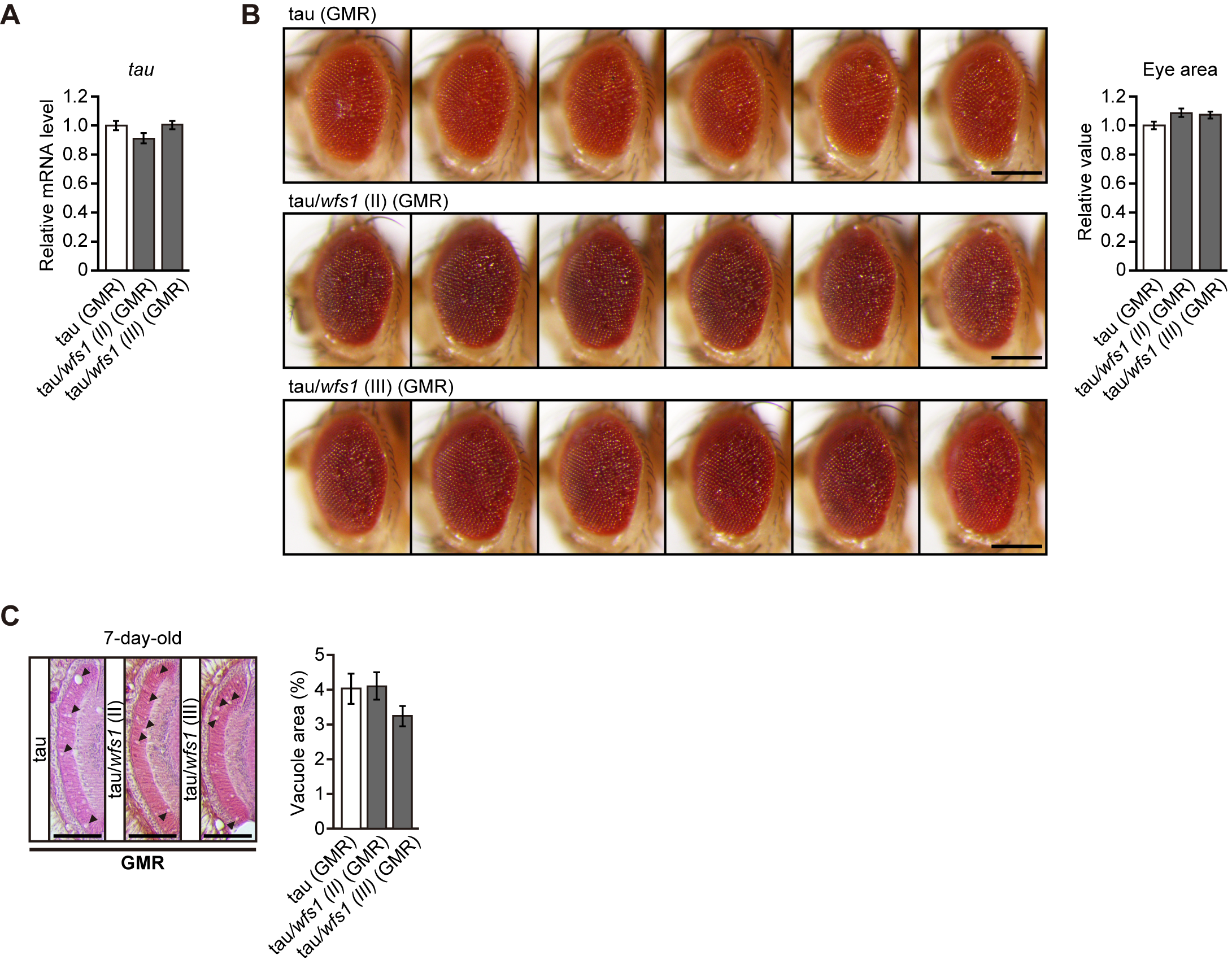

Supplement: S9 Fig — (A) mRNA levels of tau were determined by qRT-PCR. n = 4, no significant difference by one-way ANOVA with Tukey’s post hoc test. (B) Overexpression of wfs1 did not suppressed retinal degeneration induced by ectopic overexpression of human tau. Scale bar, 200 μm. (C) Overexpression of wfs1 did not suppress axon degeneration in the lamina caused by ectopic overexpression of human tau. Representative images show the lamina in paraffin-embedded head section with hematoxylin and eosin (HE) staining from 7-day-old flies. Scale bars: 200 μm. Percentages of vacuole areas in the lamina (indicated by arrowheads in the images) are shown. n = 12 hemispheres, no significant difference by one-way ANOVA with Tukey’s post hoc test. Genotypes and ages of flies are described in S1 Table. (TIF) [file pgen.1007196.s010.tif]

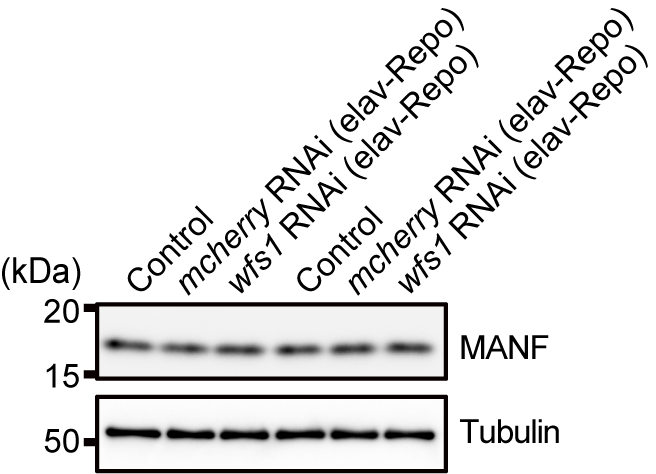

Supplement: S10 Fig — Fly heads expressing RNAi transgene for wfs1 or mcherry in both neurons and glial cells were subjected to western blotting with anti-MANF antibody. Tubulin was used as the loading control. n = 4, no significant difference. Genotypes and ages of flies are described in S1 Table. (TIF) [file pgen.1007196.s011.tif]
